# Frequency-Aware Lightweight Convolutional Neural Network for Edge-Enabled Intelligent Bearing Fault Diagnosis and Health Management

**DOI:** 10.3390/s26185861

**Published:** 2026-09-16

**Authors:** Hao Tian, Shitong Hua, Wenbiao Gui, Sijie Zou, Chenglong Wang

**Affiliations:** 1 School of Computer and Artificial Intelligence, Beijing Technology and Business University, Beijing 100048, China; 2431061007@st.btbu.edu.cn; 2 China Light Industry Key Laboratory of Industrial Internet and Big Data, Beijing Technology and Business University, Beijing 100048, China; 3 Taizhou Changxing Rail Transit Operation Management Co., Ltd., Taizhou 318000, China; wenbiaogui111@163.com (W.G.); sijiezou163@163.com (S.Z.); chenglongwang2026@163.com (C.W.)

**Keywords:** bearing fault diagnosis, lightweight convolutional neural network, frequency-domain feature, depthwise separable convolution, edge computing

## Abstract

**Highlights:**

**What are the main findings?**

- The frequency-aware lightweight convolutional neural network (FAL-CNN) achieves a 99.78% mean accuracy across four Case Western Reserve University (CWRU) operating loads (five-seed protocol, leakage-controlled splits) with only 65,122 parameters and 3.88 M multiply–accumulate operations (MACs) (7.76 M floating-point operations (FLOPs)).
- Under operating-load shift, the frequency-aware design gains 13.27 percentage points over a parameter-matched plain CNN (88.42% vs. 75.15% off-diagonal mean).

**What are the implications of the main findings?**

- On a single CPU thread, Open Neural Network Exchange (ONNX) inference takes 0.17 ms (0.27 ms end-to-end) within a 288.8 KB footprint; masking analysis confirms the multi-scale filters target fault frequency bands.
- FAL-CNN provides a compact, edge-oriented classifier with improved reliability under the evaluated distribution shifts; physical edge-device performance remains to be validated.

**Abstract:**

Intelligent fault diagnosis of rolling-element bearings is essential for predictive maintenance of rotating machinery. Although deep convolutional neural networks (CNNs) achieve high accuracy on public benchmarks, their large parameter counts and computational costs hinder deployment on resource-constrained edge devices. This paper proposes a frequency-aware lightweight CNN (FAL-CNN) that reconciles competitive accuracy with extreme efficiency. A fixed, non-trainable pipeline (fast Fourier transform, log compression, linear resampling, and min–max normalization) converts each 1024-sample vibration segment into a compact 128-dimensional spectrum, reducing the input eightfold. A frequency-aware module applies three parallel one-dimensional convolutions with kernels of 3, 7, and 15, matched to single-peak, harmonic-pair, and harmonic-family fault signatures, followed by a depthwise-separable backbone that sharply reduces parameters and floating-point operations (FLOPs). All Case Western Reserve University (CWRU) experiments use a leakage-controlled protocol with time-blocked splits, a sample-isolation gap, and five random seeds. Across four operating loads, FAL-CNN reaches a 99.78% mean accuracy with 65,122 parameters and 3.88 M multiply-accumulate operations (MACs) (7.76 M FLOPs), inferring in 0.17 ms on a single CPU thread (0.27 ms end-to-end) within a 288.8 KB Open Neural Network Exchange (ONNX) footprint. Because full-data accuracy saturates for all compact models, we evaluate where architecture matters: under operating-load shift, FAL-CNN outperforms a parameter-matched plain CNN by 13.27 percentage points (88.42% vs. 75.15%); frequency-masking and attribution analyses confirm that the multi-scale filters concentrate on fault frequency bands (a 21.91-percentage-point targeted-masking gap, roughly twice that of structural controls); under 10:1 training class imbalance, it maintains a macro-F1 above 99.2%; and on the Paderborn real damage benchmark with bearing-level nested validation, it attains (59.86 ± 18.86)% accuracy on unseen bearings, the best mean among the compared pipelines, although the difference from the parameter-matched baseline is not statistically significant. These results position FAL-CNN as a compact, edge-oriented classifier whose measured feasibility is currently limited to the development CPU; physical edge-device profiling remains required. Its demonstrated value lies in reliability under the evaluated distribution shifts rather than in saturated benchmark accuracy.

## 1. Introduction

Rolling-element bearings are among the most critical and failure-prone components in rotating machinery, including motors, gearboxes, wind turbines, railway traction systems, and aerospace actuators [1,2]. A localized defect on the inner race, outer race, or rolling element, or on the cage, progressively degrades the dynamic behavior of the machine and excites characteristic fault frequencies that are modulated into the measured vibration and acoustic signals [3,4]. An undetected bearing fault can propagate to catastrophic machine breakdown, unplanned production downtime, and, in safety-critical settings, even personnel hazards [2]. Consequently, reliable, timely, and automated bearing fault diagnosis is important for condition-based maintenance and the resilient operation of modern industrial systems.

The economic stakes are considerable. Unplanned downtime in discrete and continuous manufacturing is routinely estimated to cost from tens of thousands to over a million US dollars per hour depending on the sector, and bearing-related failures account for a substantial fraction of motor and gearbox breakdowns [2]. Even a modest improvement in early fault detection can therefore yield large savings by converting reactive repairs into scheduled maintenance windows. This economic pressure is precisely what motivates shifting intelligence from the cloud to the asset itself: a model that runs next to the machine can raise an alarm within milliseconds of a fault onset, without depending on network connectivity or centralized servers.

The cloud-vs.-edge trade-off deserves emphasis. A cloud-centric architecture streams raw vibration to a remote server for inference, which introduces network latency, bandwidth cost, and a single point of failure that is unacceptable for safety-critical assets. An edge-centric architecture, by contrast, keeps both data and inference on the device, preserving privacy, eliminating round-trip latency, and continuing to function during network outages. The principal obstacle to edge deployment is precisely the computational weight of modern diagnosis models, which is the problem this paper addresses.

For decades, bearing fault diagnosis has rested on signal processing pipelines built by domain experts. Classical methods such as envelope demodulation, spectral kurtosis, empirical mode decomposition, and wavelet transforms first extract handcrafted features, including fault characteristic frequencies, kurtosis, or band-energy ratios, and then feed them into shallow classifiers such as support vector machines or decision trees [1,4]. Although effective under stationary, well-separated, and high signal-to-noise conditions, these approaches are notoriously sensitive to environmental noise, load variation, and the correctness of prior knowledge about the machine geometry. They also struggle to generalize across different machines and operating regimes because the feature extractor must be re-engineered for every new installation [5].

The rise of deep learning has shifted the paradigm toward end-to-end data-driven diagnosis, in which a neural network learns discriminative representations directly from raw or minimally processed vibration signals, freeing the engineer from manual feature design [6]. One-dimensional convolutional neural networks (1D-CNNs) have become the de facto backbone thanks to their ability to capture local, translation-invariant patterns in vibration sequences [5,7]. Landmark architectures such as Deep Convolutional Neural Networks with Wide First-layer Kernels (WDCNN) [5], Convolution Neural Networks with Training Interference (TICNN) [7], and deeper residual networks [8] consistently report near-perfect accuracy on the Case Western Reserve University (CWRU) benchmark [3]. More recently, vision transformers and their one-dimensional variants have pushed accuracy even higher on curated test sets [9,10,11,12,13].

Despite these striking benchmark numbers, a persistent and practically important gap remains between reported accuracy and industrial deployability. State-of-the-art models tend to be large. In our own re-implementation, a 1D-ResNet-18 variant exceeds 3.8 M parameters and 21.79 M MACs (43.58 M FLOPs), while a one-dimensional vision transformer consumes 169 M reported operations [9]. Such budgets may hinder execution on microcontrollers, industrial edge gateways, and battery-powered wireless sensor nodes, whose memory, latency, and energy constraints vary substantially across platforms [14,15,16]. In real plants, a fault-diagnosis model embedded next to a motor must infer continuously and in real time, on a CPU-class processor, without exhausting the device’s primary compute and energy budgets that are simultaneously needed for control, communication, and logging [17,18].

This tension has motivated a growing body of work on lightweight diagnostic models. The dominant strategy borrows from mobile computing: depthwise-separable convolutions [14,15], channel shuffling [19], and network pruning or distillation [20,21,22] compress models at the cost of a small accuracy drop. However, most existing lightweight designs still operate on the raw long-time series, typically 1024 or more samples per segment, where the input itself, rather than the network, dominates the compute. We argue that a complementary and often overlooked lever is the input representation: a carefully engineered spectral front-end can shrink the sequence length by an order of magnitude before the network ever sees it, without sacrificing the fault information required for classification.

The physical basis for this compression is well established in rotating-machinery diagnostics. Beyond frequency concentration itself, the discriminative content of bearing signals may appear at different spectral granularities and sensing modalities. Recent studies on generalized multi-scale formal contexts, multi-stream and cross-level feature fusion, dual-graph spatiotemporal modeling, adaptive graph-attention time-series prediction, feature-mode matching, and cross-modal mutual-assistance fusion show that scale selection, representation matching, and feature fusion are important for improving classification, prediction, localization, and detection performance in complex sensing scenarios [23,24,25,26,27,28]. Meanwhile, recent predictive-maintenance studies on continuous remaining useful life (RUL) trajectory modeling and multi-mode degradation assessment further indicate that vibration/acoustic monitoring should be interpreted within a broader health management workflow rather than as an isolated classification task [29,30]. A localized defect produces a periodic impact as the rolling element strikes it once per revolution, and these impacts excite a structured spectrum in which the characteristic fault frequency and its harmonics appear as discrete lines, flanked by modulation sidebands [1,4]. In contrast, the raw time-domain waveform spreads this same information across thousands of samples interleaved with broadband noise, shaft-order components, and transient disturbances. The frequency domain is therefore a more parsimonious view: the information that discriminates one fault type from another is concentrated in a relatively small number of spectral bins, whereas the time-domain signal requires a long receptive field to accumulate the same evidence. This concentration is precisely why classical envelope and spectral-kurtosis methods were so effective, and it is the property our fixed spectral front-end exploits to reduce the input length eightfold. By coupling this compact representation with a learnable multi-scale filter, we enable the network to spend its limited capacity on the bins that matter, rather than on recovering structure that the Fourier transform already makes explicit.

In this work, we propose the Frequency-Aware Lightweight CNN (FAL-CNN), a system-level co-design that jointly optimizes the input representation and the network architecture for edge deployment, with each design choice grounded in the physics of bearing faults rather than assembled from generic components. We frame the model as a compact classifier for bearing fault diagnosis and evaluate its effectiveness through controlled comparisons covering limited labels, class imbalance, load variation, raw-signal noise, and unseen bearing conditions. Our central thesis is that bearing faults are most cleanly expressed in the frequency domain, where each defect type leaves a characteristic energy distribution, and that a compact, fixed spectral descriptor is both sufficient for classification and dramatically cheaper to process than the raw waveform. We use two engineering targets to characterize implementation compactness on the measured development platform: single-sample inference latency below 2 ms on one central processing unit (CPU) thread and a serialized model size below 1 MB. These measurements are feasibility indicators rather than validation on a physical edge board. The specific contributions are summarized as follows:

(1) We propose a system-level co-design for edge bearing fault diagnosis whose contribution is not any single module but the synergy among three coupled choices: an extreme 8× spectral compression, a physically matched multi-scale filter bank, and a depthwise-separable backbone. Jointly, they shrink both the input length and the per-layer cost, which is why the full model reaches only 65,122 parameters and 3.88 M MACs (7.76 M FLOPs).
(2) We provide a quantitative, physically grounded rationale for the spectral front-end and the kernel sizes. We show that the 128-dim compression retains the CWRU fault frequencies with a comfortable margin and that the three kernels (3, 7, 15) correspond to spectral receptive fields of approximately 141, 328, and 703 Hz, matching a single characteristic frequency peak, the peak plus its first harmonic, and the peak plus multiple harmonics of the bearing fault spectrum, respectively.
(3) We adopt a depthwise-separable backbone that compounds the 8× input reduction with a channel-wise factorization, reducing the computational burden for resource-constrained inference hardware; target-device feasibility still requires platform-specific measurement.
(4) We validate the approach on the CWRU 12 kHz Drive End dataset across four operating loads under a leakage-controlled time-blocked protocol with five-seed repetition, achieving a mean accuracy of 99.78%. We export the model to ONNX and report 0.17 ms single-sample inference latency, 0.27 ms preprocessing-plus-inference latency, and a 288.8 KB footprint on the development CPU. These results demonstrate implementation feasibility on the measured platform but do not constitute physical edge-device validation.
(5) Through controlled comparisons against parameter-matched and capacity-scaled baselines, ablations with frequency-masking and attribution evidence, cross-load transfer, class-imbalance, raw-signal noise, and unseen bearing (Paderborn) evaluations, we delineate the conditions under which the frequency-aware design provides measurable reliability gains over plain convolutional stacks of equal size and the conditions under which it does not.

The remainder of this paper is organized as follows. Section 2 reviews related work on signal-based, deep-learning-based, transformer-based, and lightweight fault diagnosis. Section 3 details the proposed spectral front-end and network architecture, including complexity analysis. Section 4 presents the experimental setup, main results across loads, controlled comparisons, ablations with frequency-domain evidence, deployment profiling, evaluation under limited labels and class imbalance, robustness studies, external validation on real damage data, and discussion. Section 5 concludes the paper and discusses limitations and future directions.

## 2. Related Work

The literature on bearing fault diagnosis is broad and has evolved through three overlapping waves: classical signal processing, deep convolutional models, and, most recently, large transformer-based and lightweight edge-oriented architectures. This section situates our work within these lines of work. We deliberately separate the question of how accurately a model can classify faults from the question of whether it can run on an edge device, because the two have historically been treated separately and only recently converged into the field of TinyML for industrial diagnosis [16,17].

### 2.1. Signal Processing and Classical Methods

Before the deep learning era, bearing diagnosis was dominated by model-based and signal processing techniques. The foundational theory relates defect geometry to characteristic fault frequencies (inner race, outer race, ball spin, and cage frequencies) that appear as spectral lines or sidebands in the vibration spectrum [1]. Envelope analysis and spectral kurtosis were developed to extract these fault-induced impulses from heavy background noise [4]. Time–frequency tools such as the wavelet transform and empirical mode decomposition further localized faults in both time and frequency [31]. Although these methods offer physical interpretability and require no training data, they require accurate prior knowledge of the machine’s geometry and operating speed, and their handcrafted features rarely transfer across machines or loads [32]. Their sensitivity to noise and the need for expert tuning make them brittle in real industrial settings, which is the primary reason data-driven methods have gained prominence. Nevertheless, classical methods remain valuable as a source of interpretability and as weak priors for hybrid systems. Several recent studies combine physics-based fault frequency knowledge with learned feature extractors to improve robustness under speed variation [33]. Our work targets a complementary axis, extreme efficiency, and therefore relies on a fixed spectral descriptor rather than learned physics, leaving hybrid physics-informed lightweight models as a promising direction for future work.

### 2.2. Deep Convolutional Models

Deep learning removed the need for manual features. Zhang et al. proposed WDCNN, a seven-layer 1D-CNN with a large first kernel that learns directly from raw signals and achieves high accuracy under varying loads [5]. Zhang et al. introduced TICNN which adds a trainable denoising front-end and a competitive layer to improve robustness to noise [7]. To address cross-domain transfer, Lu et al. developed a deep separable network (DSN) that aligns feature distributions between a labeled source machine and an unlabeled target [8]. Subsequent studies explored attention mechanisms [34,35], wavelet- and interpretable kernels [36,37], multi-scale and adversarial designs [38,39,40], and self-supervised pre-training [41]. A recent survey summarizes the breadth of CNN-based diagnosis and notes that, while accuracy on public benchmarks has saturated near 99%, the dominant open problem has shifted from accuracy to efficiency and generalization [32]. A common weakness of these CNN models is that accuracy improvements have frequently come at the cost of growing depth and width, pushing the compute budget upward. While this trend improved benchmark numbers, it also distanced the models from the resource envelope of edge hardware. Our work deliberately inverts this trend by asking how small a network can be made before accuracy collapses and shows that the collapse point is far below the prevailing designs.

A parallel line of work, closer to ours, preprocesses vibration into the frequency or time–frequency domain before feeding a CNN. Fast Fourier transform (FFT)-based magnitude spectra, envelope spectra, mel-like filter banks, and wavelet scalograms have all been used as compact network inputs, on the premise that fault information is more accessible in these transformed representations than in the raw waveform [33,36]. This practice has two broad benefits: it shortens the effective sequence length, and it makes the network less sensitive to phase and to small time-domain perturbations. However, most of these approaches treat the spectral transform either as a fixed one-time preprocessing step whose exact configuration is rarely ablated or as a learned module that reintroduces parameters and training cost. Our contribution differs in making the spectral front-end a first-class, explicitly analyzed design decision: we fix a minimal, non-learnable pipeline, justify each stage (log compression, resampling, normalization), and quantify through ablations how much the spectral compression itself contributes to efficiency vs. accuracy.

Two very recent works are particularly close in spirit and warrant explicit differentiation. MSFF-Net [42] converts vibration and acoustic signals to the frequency domain via FFT and fuses modality-specific features through a lightweight 1D-CNN, reporting 99.73% accuracy and 94.69% under the 20-shot protocol; its central contribution is multi-sensor fusion, and it treats the FFT as a fixed preprocessing step without analyzing the compression ratio or the kernel–frequency correspondence. LTFANet [43] processes raw one-dimensional waveforms with multi-scale depthwise-separable convolutions, a learned frequency branch, and channel attention, reaching 97.20% on CWRU-10 with 0.020 M parameters, and further relies on knowledge distillation, pruning, and TensorRT acceleration for its final efficiency. FAL-CNN differs from both on three concrete axes. First, we fix an extreme 8× compression (1024 to 128 dimensions) and verify its adequacy against the fault frequency geometry, whereas MSFF-Net leaves the transform unablated and LTFANet retains the raw 1024-length input with a learned frequency path. Second, our multi-scale kernels are physically matched to the single-peak, harmonic-pair, and harmonic-family scales of the fault spectrum (Section 3.3), rather than being a generic multi-scale stack. Third, our evidence for the design comes from controlled stress conditions rather than saturated full-data accuracy: under operating-load shift, the frequency-aware model outperforms a parameter-matched plain CNN by 13.27 percentage points (Section 4.8), and frequency-masking plus attribution analyses show that the multi-scale filters concentrate on fault frequency bands (Section 4.5). We do not claim that FFT, multi-scale convolution, or separable convolution are novel in themselves; the contribution is their system-level co-design, the quantitative grounding of each choice in the physics of bearing faults, and the leakage-controlled, multi-seed protocol used to validate it.

### 2.3. Transformers and Large Models

Inspired by the success of transformers in vision [10,11], recent work adapts them to one-dimensional vibration. Xu and Zhang proposed a one-dimensional vision transformer (1D-ViT) that patches the signal and applies self-attention, reaching 99.9% on CWRU [9]. More recent 2024–2025 studies combine time–frequency decomposition with vision transformers (e.g., TFDAViT, GTFE-NET) to set new accuracy records [12,13,44]. Although these models push the accuracy ceiling, their parameter and MAC counts are typically one to two orders of magnitude larger than what an edge device can afford, and their quadratic attention cost grows with sequence length [33]. For the bearing diagnosis task, where the useful discriminative content is concentrated in a compact spectral signature, the representational overhead of a full transformer is difficult to justify on an edge device. From an edge perspective, the attention mechanism is doubly expensive: it is both parameter-heavy and quadratic in sequence length. For a 1024-sample waveform, this quadratic cost is severe, and even patched variants retain substantial overhead. This observation motivates replacing the long raw sequence with a short spectral descriptor, which not only shrinks the convolutions but also tames any attention-like component should one be added later, because the sequence length is reduced eightfold before any such module is applied.

### 2.4. Lightweight and Edge-Oriented Models

The mobile deep learning community offers a broad toolbox for compression: depthwise-separable convolution [15], MobileNet and its width multipliers [14,45], ShuffleNet [19], SqueezeNet [20], and efficient architectures such as EfficientNet [46]. These principles have been ported to fault diagnosis: a growing body of 2021–2024 work reports lightweight 1D-CNNs [47,48,49,50], separable convolutions [51,52], channel- or self-attention enhancements [35,53,54], knowledge distillation [55,56], and surveys of the field [17,32]. Model compression techniques, including pruning [21], quantization [57,58,59], and knowledge distillation [22,60] further shrink deployed models, while studies of edge monitoring and edge-intelligent diagnosis [16,18] examine on-device inference. Recent work also addresses noise-robust diagnosis [61], reinforcement-learning-based feature enhancement [62], and limited-sample attention models [63]. Nevertheless, two limitations persist. First, most lightweight designs retain raw long inputs, leaving a large, fixed cost [33,61]. Second, few works provide a complete edge-deployment profile (latency, footprint, and an industry-standard inference engine), which is essential for practical adoption [17]. This paper targets both gaps by co-designing a spectral front-end and a separable backbone and by validating the exported model on ONNX Runtime. Despite the active research above, direct comparisons of edge readiness remain rare. Many papers report accuracy and parameter counts but stop short of measuring latency on a real inference engine or model size after export. Without these measurements, claims of “lightweight” are difficult to benchmark. Our contribution is therefore not only a compact architecture but also a reproducible development-CPU implementation profile using ONNX Runtime; physical edge-device validation remains necessary.

## 3. Materials and Methods

### 3.1. Problem Formulation and Signal Preprocessing

Experimental platform: Model training and evaluation were performed on a workstation equipped with a 12th Gen Intel Core i9-12900H CPU (Intel Corporation, Santa Clara, CA, USA) and an NVIDIA GeForce RTX 3060 Laptop GPU (NVIDIA Corporation, Santa Clara, CA, USA). The software environment consisted of Python 3.10.19, PyTorch 2.2.2 with CUDA 12.1, and NumPy 1.24.3. The trained model was exported to the ONNX format using opset 13. Deployment latency was measured on the CPU using ONNX Runtime 1.19.2 with the CPUExecutionProvider; both the intra-op and inter-op thread counts were set to one.

We formulate bearing fault diagnosis as a supervised multi-class classification problem. The objective is to map a short vibration segment to one of C=10 categories: one healthy class (Normal) and nine fault classes spanning three fault locations (inner race (IR), ball (B), outer race (OR)) and three severity levels (7, 14, and 21 mils of defect diameter). Each raw signal is the drive end (DE) acceleration recorded at 12 kHz. The multi-class formulation is chosen over binary healthy/fault detection because it supports condition monitoring and remaining-useful-life planning, where knowing the fault location and severity is as important as detecting its presence.

$$L=1024\;x\in R^{1024}$$

From each raw recording, we extract fixed-length segments using a sliding window of length samples with a stride of 256 samples (75% overlap). The overlap increases the number of training samples and improves the robustness of stochastic gradient descent without materially changing the class distribution. Each segment is standardized by Z-score normalization. The train/validation/test partitioning is performed at the level of continuous-record time blocks before window extraction, with a 1024-sample isolation gap at every block boundary, so that no window’s raw samples are shared across splits (Section 4.1).

(1) $$\begin{array}{c} \hat{x}=\left(x-\mu \right)/\left(\sigma +\varepsilon \right) \end{array}$$

where μ and σ are the segment mean and standard deviation, and ε is a small constant for numerical stability. This per-segment normalization is robust to sensor gain, load-induced amplitude shifts, and slow drift across operating conditions, and it keeps the subsequent network in a favorable dynamic range. The normalization statistics are computed within each segment and therefore can be reproduced exactly on an edge device without storing population statistics.

### 3.2. Frequency-Domain Feature Transform

**Figure 1:**
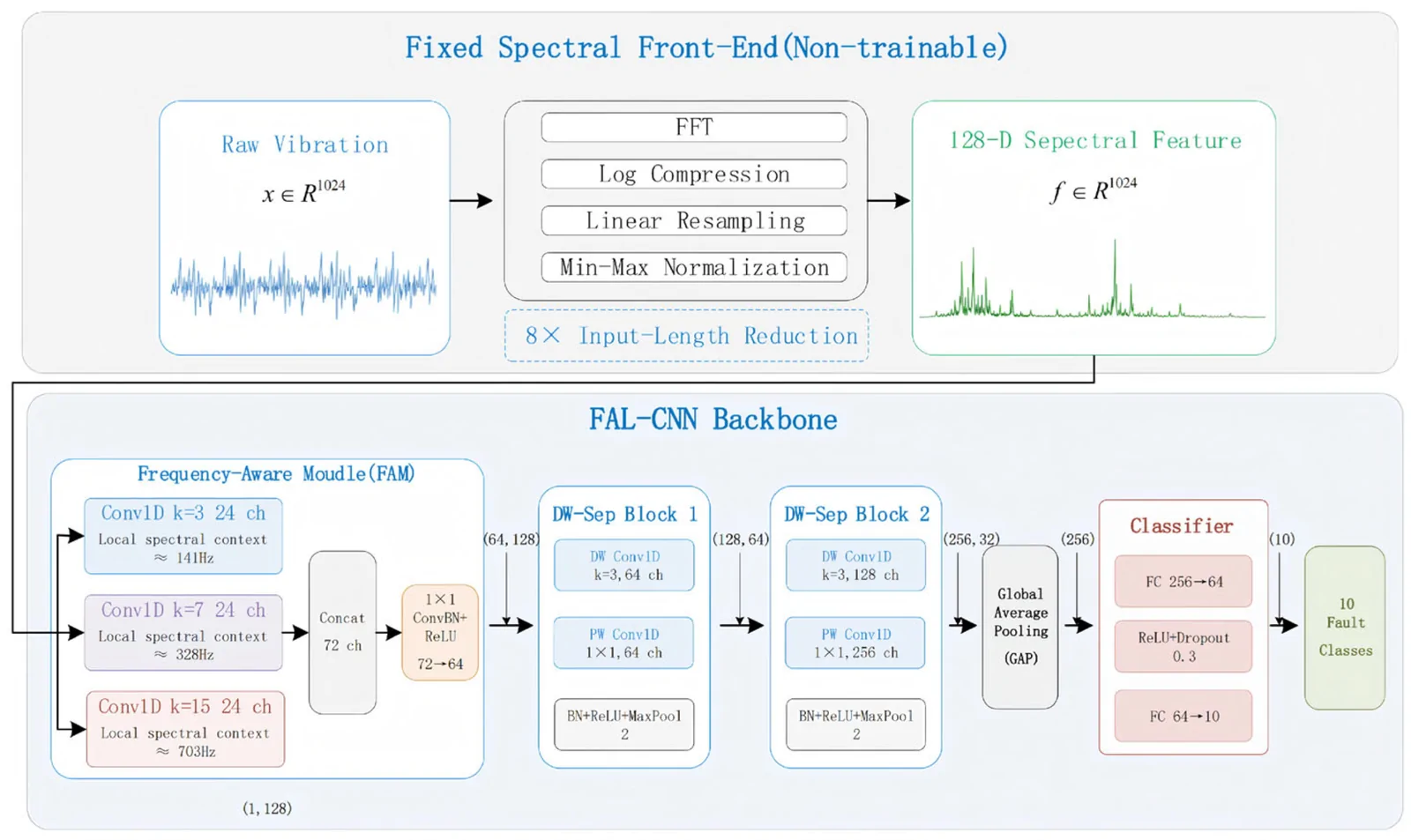
Overall framework of frequency-aware lightweight convolutional neural network (FAL-CNN). The upper non-trainable spectral front-end transforms each 1024-sample vibration segment into a 128-dimensional spectral feature via fast Fourier transform (FFT), log compression, resampling, and min–max normalization. The lower trainable backbone comprises the frequency-aware module, two depthwise-separable blocks, global average pooling, and the classifier.

Rather than feeding the 1024-sample time series directly to the network, we map it to a compact spectral representation through a fixed, non-trainable pipeline (Figure 1):

(1) FFT and positive frequency selection. We compute the real fast Fourier transform of the normalized signal $\hat{x}$ and retain the non-negative frequency bins, yielding $N_{f}=513$ bins and the magnitude spectrum $s\in R^{513}$.
(2) Log compression. We apply $\hat{S}=log\left(1+s\right)$, which compresses the wide dynamic range of vibration spectra and emphasizes small-but-discriminative peaks [4].
(3) Linear resampling. The 513-bin spectrum is resampled to a fixed D=128 dimensions through linear interpolation, yielding a resolution-invariant feature regardless of the original sampling rate.
(4) Per-segment min–max normalization. Each 128-dim vector is scaled to [0, 1], improving training stability and making the network insensitive to absolute spectral scale.

**Figure 2:**
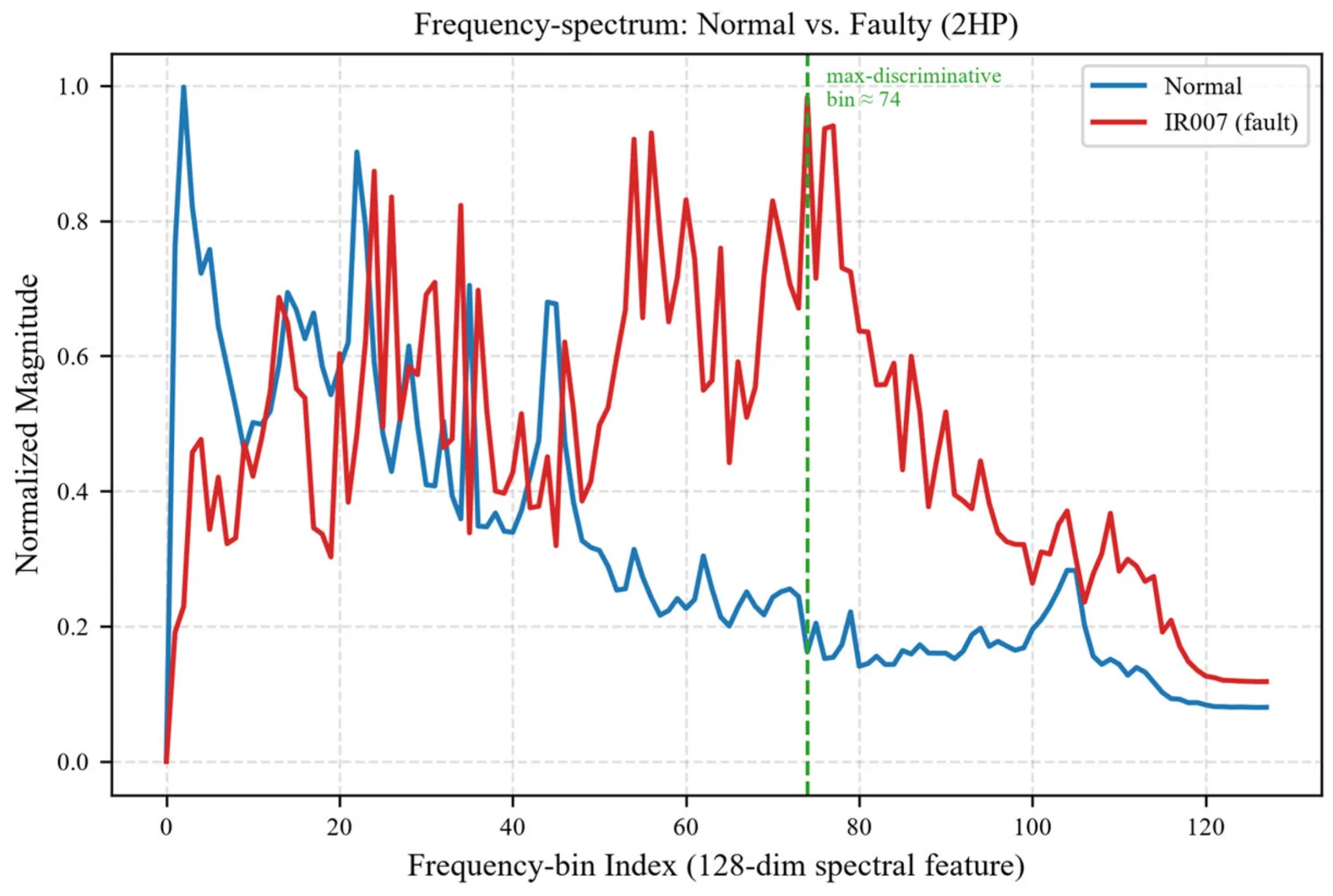
Average normalized spectra of healthy and faulty (IR007) signals ((2 horsepower (HP) load). Faults introduce pronounced energy in distinct frequency bins, which the frequency-aware module is designed to capture.

The choice of D=128 is a deliberate trade-off. It is large enough to retain the separation between the fundamental fault frequencies (which for CWRU lie below approximately 1.2 kHz, i.e., within the first ~100 bins of the positive spectrum) and their harmonics, yet small enough to keep the downstream network tiny. At the 12 kHz sampling rate, the Nyquist frequency is 6 kHz, so the 513 positive frequency bins span 0–6 kHz at roughly 11.7 Hz per bin; the 128 resampled bins therefore still resolve the CWRU characteristic frequencies (which fall below 1.2 kHz) with comfortable margin. Because the transform is linear interpolation followed by affine scaling, it can be computed on an edge device with a precomputed resampling matrix and a few arithmetic operations per sample, adding negligible latency. As visualized in Figure 2, healthy and faulty spectra are clearly separated: faults introduce pronounced energy in specific bins that align with their characteristic frequencies. Because this transform is fixed and has no learnable parameters, it adds neither parameters nor training cost and can be implemented with a handful of CPU instructions on an edge device.

The log compression and min–max normalization stages deserve brief justification, as both directly affect the discriminability and numerical behavior of the feature. The raw magnitude spectrum of a bearing vibration spans several orders of magnitude, with dominant shaft-order and resonance peaks that can dwarf the fault lines of interest; the logarithm suppresses this dynamic range so that low-energy but diagnostically informative peaks are not lost in the quantization floor. Per-segment min–max normalization then bounds the feature to [0, 1], which stabilizes gradient descent and removes any dependence on the absolute sensor gain or the global signal amplitude. Both operations are monotonic and therefore preserve the ordinal structure of the spectrum, meaning that the relative prominence of a fault line over its neighborhood, the very cue that separates healthy from faulty states, is retained. Because no learnable statistics are involved, the entire front-end is deterministic and identical at training and deployment time, eliminating any train–serve skew. We denote the resulting 128-dimensional spectral feature as $f\in R^{128}$.

### 3.3. Frequency-Aware Module

Fault characteristic frequencies of inner-race, ball, and outer-race defects occupy different regions of the spectrum and exhibit different bandwidths. To capture these multi-scale patterns, the frequency-aware module (FAM) applies three parallel one-dimensional convolutions to f with kernel sizes k ∈ 3, 7, 15 and 24 channels each. The outputs are concatenated along the channel axis (72 channels) and fused by a 1 × 1 convolution that projects them to 64 channels, followed by batch normalization and a rectified linear unit (ReLU) non-linearity:

(2) $$\begin{array}{c} h=\mathrm{ReLU}\left(BN\left({Conv}_{1\times 1}\left[Concat\left({Conv}_{3}f,{Conv}_{7}f,{Conv}_{15}f\right)\right]\right)\right) \end{array}$$

The heterogeneous kernels act as multi-resolution spectral filters: the small kernel (3) responds to narrow peaks, the medium kernel (7) to moderate bands, and the large kernel (15) to broad spectral envelopes. This design is motivated by the observation that different fault types leave signatures at different frequency resolutions and that fusing them early provides the backbone with a multi-scale spectral representation. The parallel structure also keeps the parameter count low, because each branch is shallow and narrow.

The three specific kernel sizes are not arbitrary but are matched, by construction, to the frequency geometry of the spectral feature and to the structure of the bearing fault spectrum. At the 12 kHz sampling rate, the 1024-point FFT has a native resolution of approximately 11.7 Hz per bin, and the 128-dim resampled spectrum spans the 0–6 kHz band, so each spectral bin covers approximately 46.9 Hz. The three kernels therefore have spectral receptive fields of approximately 141, 328, and 703 Hz. These three scales map directly onto the multi-scale anatomy of the fault spectrum. Taking the 2 horsepower (HP) condition as a concrete example, the outer-race, inner-race, and ball-spin characteristic frequencies each manifest as a single dominant spectral line: the k = 3 kernel, with a 141 Hz field of view, isolates one such line together with its immediate spectral leakage without mixing it with a neighboring fault line. The k = 7 kernel (328 Hz) spans a characteristic frequency together with its first harmonic; for instance, a BPFO at 105 Hz and 2 × BPFO at 210 Hz are separated by 105 Hz, which captures the harmonic pairs that most cleanly separate healthy from faulty states. The k = 15 kernel (703 Hz) spans a characteristic frequency together with its first several harmonics (e.g., BPFO at 105, 210, and 315 Hz) and the modulation sidebands spaced by the shaft rotation frequency (approximately 29 Hz), capturing the broad spectral envelope that characterizes an impulsive defect. In short, the three kernels form a compact, hand-designed filter bank whose receptive fields are tuned to the single-peak, harmonic-pair, and harmonic-family scales of the bearing fault spectrum, rather than being a generic Inception-style multi-scale choice. This physical grounding is examined directly through frequency-masking and attribution analyses in Section 4.5, and its practical benefit appears under operating-load shift (Section 4.8); it is a concrete point of difference from methods that adopt multi-scale convolution without relating the kernel sizes to the fault physics.

### 3.4. Depthwise-Separable Backbone

After FAM, the 64-channel feature map is processed by two depthwise-separable convolutional blocks that progressively expand the channel width to 128 and then 256. Each block consists of:

(1) A depthwise convolution (groups = input channels) that filters each channel independently;
(2) A pointwise 1 × 1 convolution that mixes channels;
(3) Batch normalization and a ReLU activation;
(4) A max-pooling layer that halves the sequence length.

$$\frac{1}{Cout}+\frac{1}{k^{2}}$$

Depthwise-separable convolution factorizes a standard convolution into depthwise and pointwise operations. Let Cin, Cout, k, and L denote input channels, output channels, kernel size, and sequence length. A standard convolution costs CinCoutkL multiply-accumulate operations (MACs), whereas a depthwise-separable block costs CinkL + CinCoutL, a reduction of approximately [15]. Throughout this paper, one MAC denotes one multiplication followed by one accumulation; FLOPs are reported as 2 × MACs by counting the multiplication and addition separately. A global average pooling (GAP) layer collapses the spatial dimension, and a classifier head maps the 256-dim vector to the 10 classes through two fully connected layers (256 → 64 → 10) with dropout (*p* = 0.3) on the hidden layer to mitigate over-fitting given the small parameter budget. To make the data flow concrete, the tensors evolve as follows:

$$\left(1,128\right)\overset{\mathrm{FAM}}{\to }\left(64,128\right)\overset{\mathrm{Block}\;1}{\to }\left(128,64\right)\overset{\mathrm{Block}\;2}{\to }\left(256,32\right)\overset{\mathrm{GAP}}{\to }\left(256\right)\overset{\mathrm{Classifier}}{\to }\left(10\right)$$

where the second dimension is the (halved-twice) sequence length; all operations are 1D in the frequency-bin axis. This compact topology is what keeps the parameter and MAC counts 5–60× below typical ResNet- or transformer-based alternatives.

### 3.5. Design Rationale and Forward Pass

The overall forward pass can be summarized as follows:

(3) $$\begin{array}{c} \;\;\hat{y}=Classifier\left(GAP\left(Backbone\left(FAM\left(\Phi \left(X\right)\right)\right)\right)\right) \end{array}$$

where Φ is the fixed spectral transform and all trainable parts are confined to FAM, Backbone, and Classifier. Two design decisions deserve note. First, by placing the heavy lifting of representation in the non-trainable Φ, the learnable network only needs to separate already discriminative spectral features, which is why a small model suffices. Second, the 8× reduction of the input length propagates through every subsequent layer: a convolution over a 128-length sequence costs 8× less than over a 1024-length sequence for the same channel configuration, which is the single largest contributor to the low MAC count. These choices are validated by the ablations and the frequency evidence analyses in Section 4.5.

### 3.6. Training Objective

The network is optimized with the standard softmax cross-entropy loss between the predicted class distribution ŷ and the one-hot encoded ground-truth label y:

(4) $$\begin{array}{c} \;\;\;L=-\sum_{c=1}^{C}y_{c}\log{\hat{y}}_{c} \end{array}$$

No auxiliary losses, mixup, or label smoothing are used to keep the training pipeline simple and reproducible. Gradients are computed with automatic differentiation and accumulated over mini-batches of 32 segments. Because the input dimension is only 128 and the network is shallow, a single epoch over the training set completes in a few seconds on the CPU, so the full 50-epoch budget, or the early-stopped subset thereof, is inexpensive. We deliberately avoid aggressive data augmentation (e.g., time-domain mixing or noise injection) so that the reported accuracy reflects the capacity of the architecture rather than the augmentation policy; modest augmentation is left as a straightforward future enhancement that is fully compatible with the fixed front-end.

### 3.7. Complexity Analysis

The total FAL-CNN contains 65,122 trainable parameters and 3.88 M MACs (7.76 M FLOPs), providing a compact candidate for resource-constrained inference; suitability for a specific target device requires direct profiling. A detailed complexity and deployment profile, including latency on a standard inference engine, is presented in Section 4.6. The compactness arises from two compounding factors: the 8× shorter spectral input and the depthwise-separable factorization, both of which attack the dominant terms in the convolution cost.

## 4. Experiments

### 4.1. Dataset

We use the CWRU 12 kHz Drive End bearing dataset [3], the most widely adopted benchmark for bearing fault diagnosis. It comprises four operating loads (0, 1, 2, and 3 horsepower [HP], corresponding to motor speeds of 1797, 1772, 1750, and 1730 rpm) and ten categories each: normal plus inner-race (IR), ball (B), and outer-race (OR) faults at 7, 14, and 21 mil severities. To prevent information leakage, the data are partitioned at the level of continuous recording time blocks before any window extraction: each recording is divided into chronological blocks assigned to training, validation, and test, and windows (L = 1024, stride 256) are then extracted within each block. A 1024-sample isolation gap at every block boundary removes any sharing of raw samples between adjacent blocks, and a post hoc audit verified zero cross-split window overlap. After class-balanced down-sampling, each load contains 3190 training, 660 validation, and 660 test windows, i.e., 319/66/66 per class (Table 1); the identical partitioning is shared by every compared model. We deliberately evaluate under the more realistic setting in which a separate model is trained and deployed per operating load, because an edge device installed at a given site faces essentially a single load condition; this avoids the optimism of mixed-load training while still demonstrating consistent performance across the full range of loads. The class-balanced protocol means that the validation and test sets are balanced, so a majority-class baseline would score only 10%, showing that the achieved accuracy of approximately 99.8% reflects genuine discriminative learning rather than exploitation of imbalance.

We acknowledge the known limitations of the CWRU benchmark: the signals are collected under controlled laboratory conditions with relatively low ambient noise, the faults are artificially induced, and the dataset is now more than a decade old. We retain it for three reasons. First, it remains the de facto standard against which the vast majority of published methods are reported, which enables the background comparison in Section 4.4. Second, our contribution concerns the architecture and the input representation rather than the data regime; the efficiency results (parameters, FLOPs, latency) transfer to any vibration task with a similar sampling rate. Third, to address the single-benchmark concern directly, we additionally evaluate the Paderborn real damage dataset with bearing-level splits (Section 4.9), which introduces a different acquisition platform, real damage formed by accelerated lifetime testing, and unseen bearing evaluation. Field validation on noisier industrial data remains an important direction of future work.

### 4.2. Training Setup

All models are trained with a batch size of 32 for up to 50 epochs using the AdamW optimizer [64,65] (weight decay 10^−4^) and a cosine-annealing learning-rate schedule [66]. Early stopping with patience 10 on validation loss selects the best checkpoint. Experiments run on a CPU, with 16-bit automatic mixed precision enabled when a CUDA device is available. Every CWRU result in this paper is the mean ± standard deviation over five random seeds (0, 1, 2, 3, 42) under the leakage-controlled protocol; single-seed legacy numbers are explicitly labeled wherever they are retained. The Paderborn study uses a nested protocol with three seeds inside a bearing-level five-fold split (Section 4.9). All preprocessing uses only standard, dependency-light numerical operations, and all compared models share identical data splits.

### 4.3. Main Results Across Operating Loads

**Figure 3:**
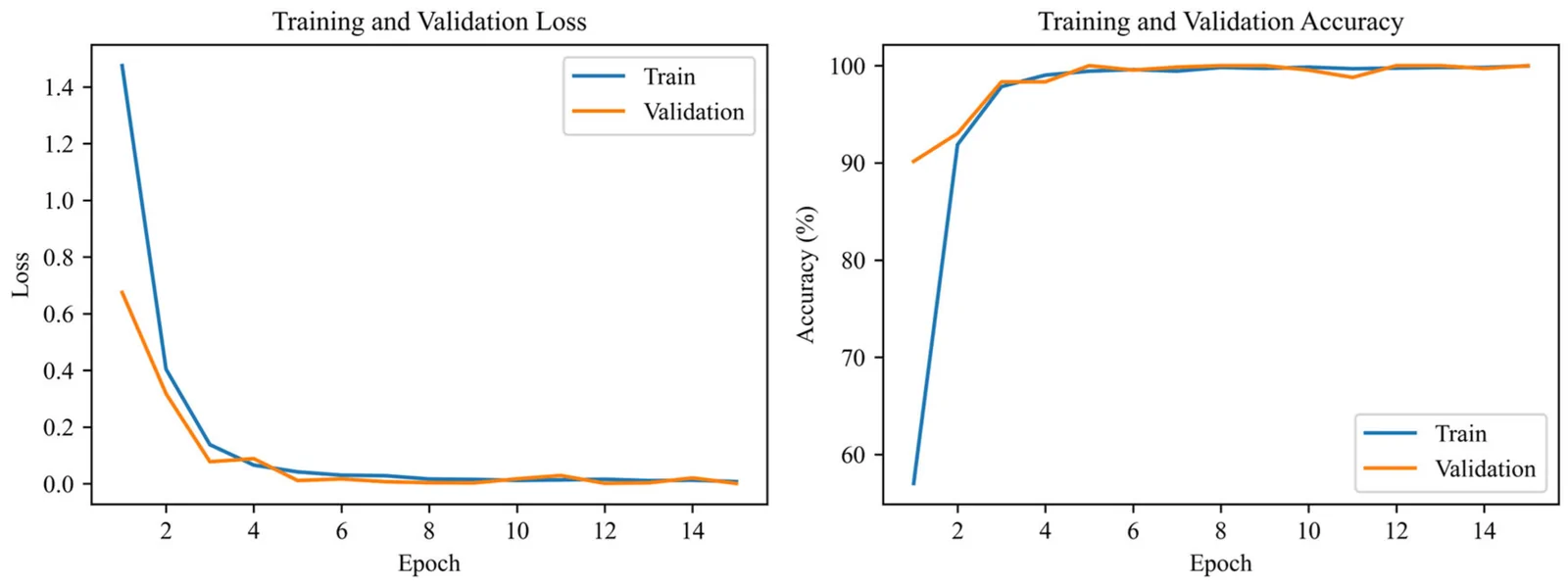
Training and validation loss and accuracy curves for the representative FAL-CNN run on the CWRU 2 HP load (seed 0). Model selection uses validation accuracy, and the test set is evaluated once after checkpoint selection.

**Figure 4:**
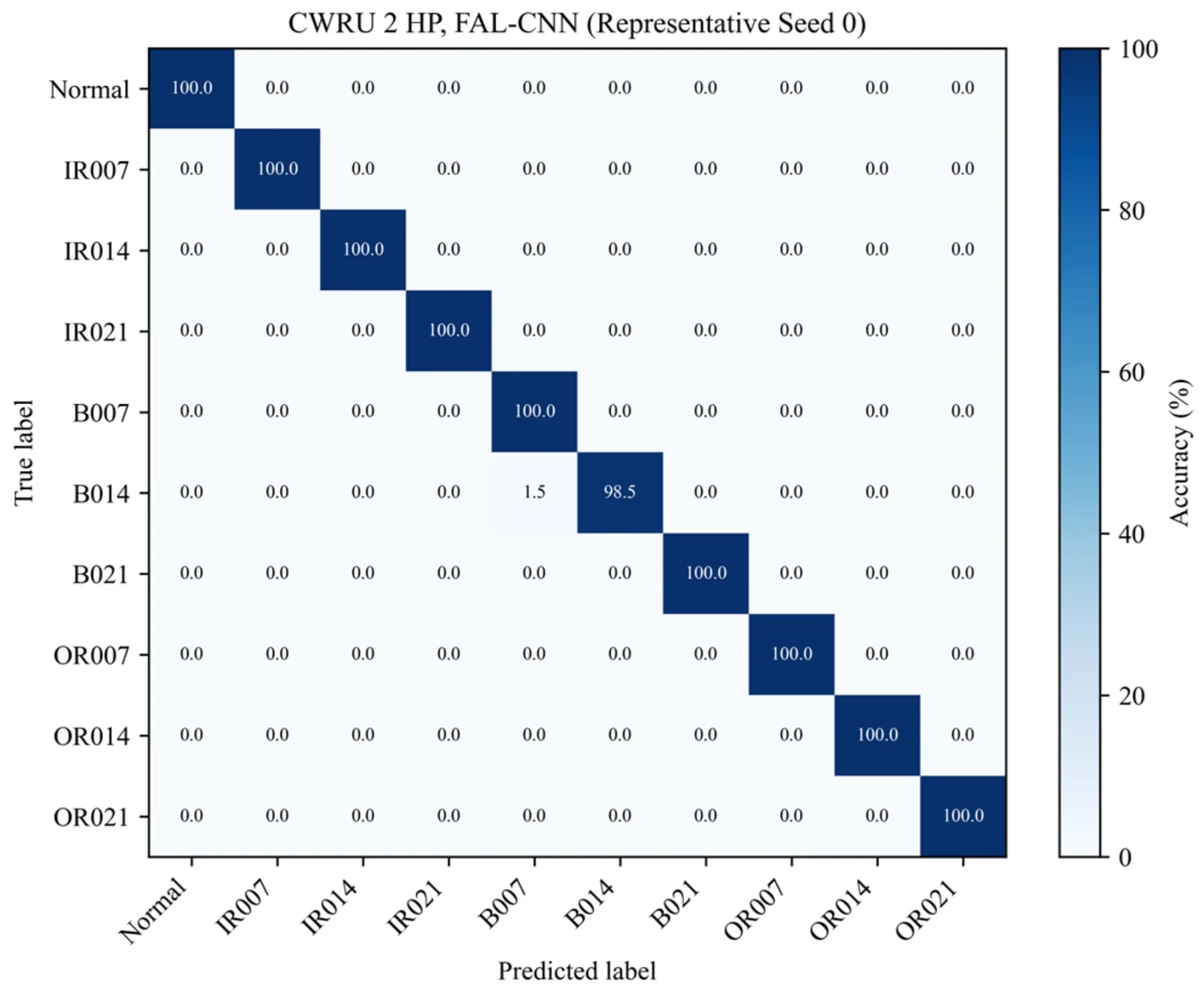
Confusion matrix of FAL-CNN on the CWRU 2 HP test set under the leakage-controlled protocol (percentages, representative seed 0). The only error is B014 classified as B007, yielding 99.85% accuracy for this run.

**Figure 5:**
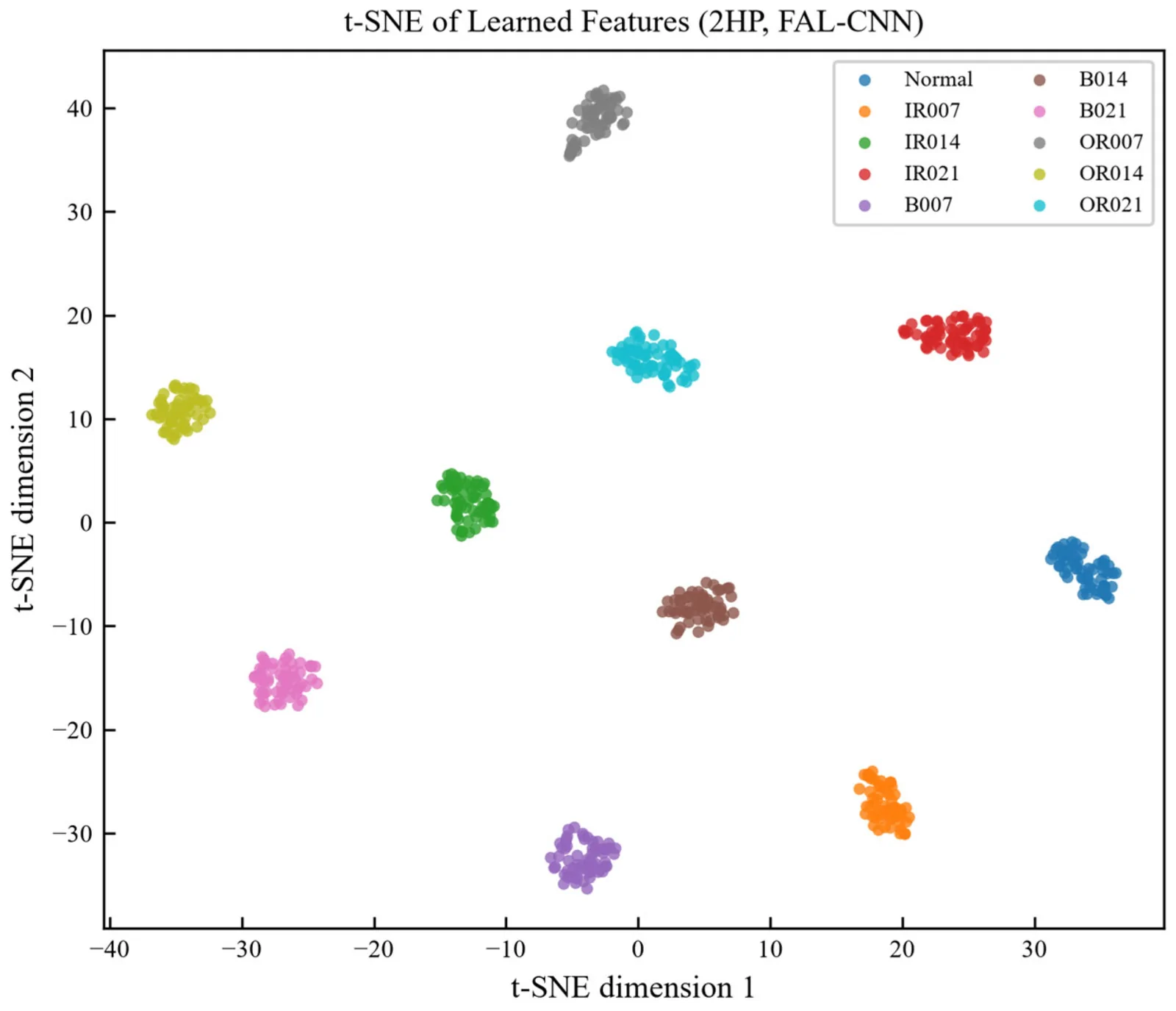
t-distributed stochastic neighbor embedding (t-SNE) visualization of the 128-dimensional spectral test features (2 HP load), colored by fault class. The ten classes form well-separated clusters, confirming the discriminability of the spectral representation.

We train and evaluate FAL-CNN independently on the four loads, repeating every training with five random seeds (0, 1, 2, 3, 42). The mean test accuracies are (99.64 ± 0.49)%, (99.76 ± 0.38)%, (99.88 ± 0.13)%, and (99.82 ± 0.13)% on the 0, 1, 2, and 3 HP loads, respectively, with a four-load mean of 99.78%; no load falls below 99.6%, and the largest seed-level standard deviation is 0.49 percentage points. The training curves (Figure 3) show rapid convergence within a few epochs and stable validation behavior without signs of over-fitting. The confusion matrix for the 2 HP load (Figure 4) contains one residual error, in which B014 is classified as B007; this is a confusion between ball-fault classes of different severities rather than between different fault locations. The t-distributed stochastic neighbor embedding (t-SNE) of the 128-dim spectral features (Figure 5) forms well-separated clusters per class, confirming that the fixed spectral front-end already provides a strongly discriminable representation even before the learnable backbone. An important practical observation is the consistency of this behavior across all four loads: the same front-end and the same architecture attain uniformly high accuracy, so a deployment team can reuse one design and one training recipe across a fleet of machines operating at different speeds, and the small, reproducible differences across loads can be anticipated during commissioning rather than discovered in the field.

### 4.4. Comparison with State-of-the-Art

**Figure 6:**
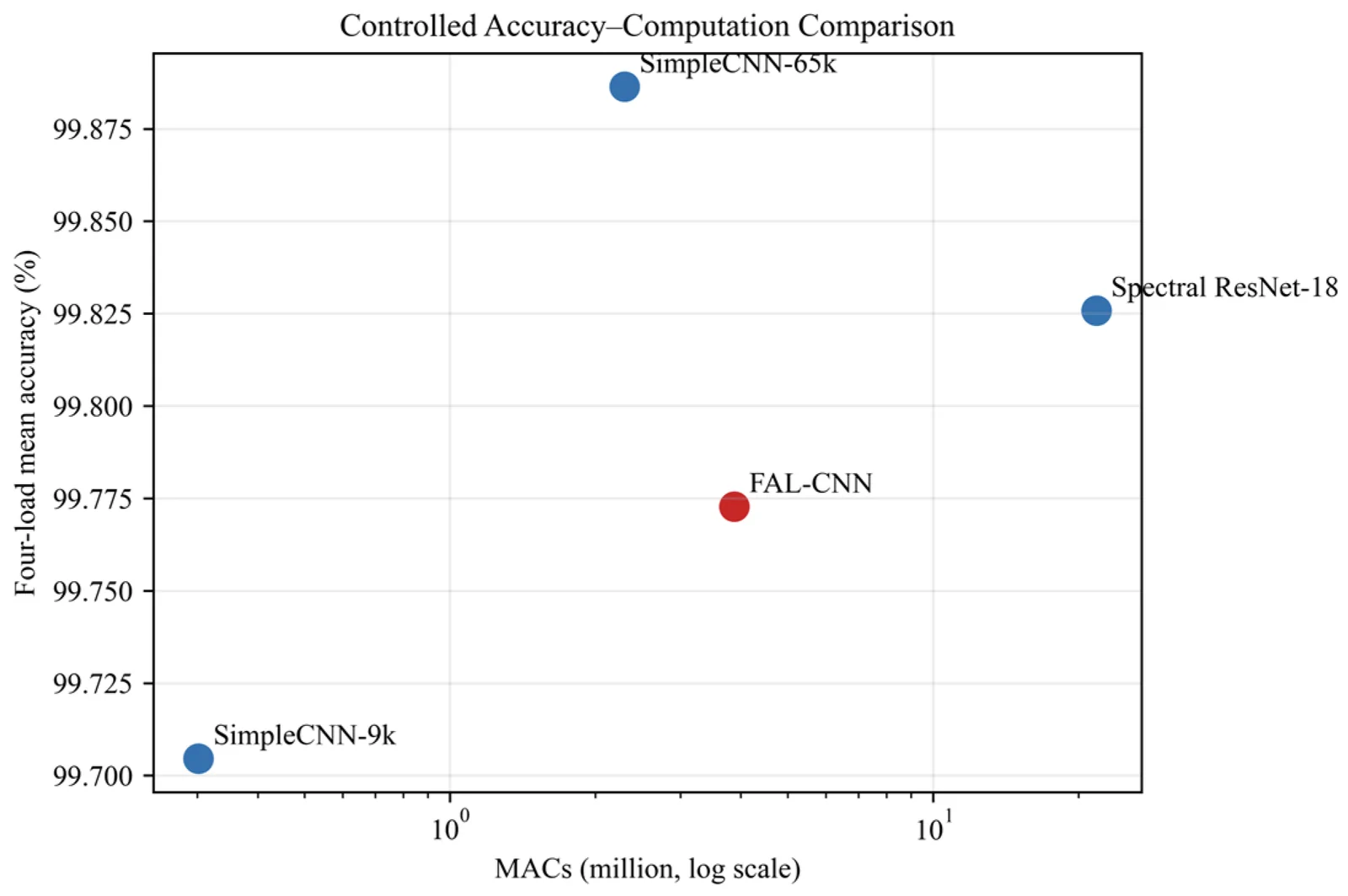
Controlled accuracy–computation comparison of the four self-run models. Accuracy is the mean across four CWRU loads and five seeds; computational cost is reported as multiply-accumulate operations (MACs) on the common 128-bin spectral input.

We compare FAL-CNN against three self-implemented baselines evaluated under identical time-blocked splits, identical inputs, and the same five seeds: a parameter-matched plain CNN (SimpleCNN-65k, 65,090 parameters), an aggressively compressed variant (SimpleCNN-9k, 8714 parameters), and a 1D-ResNet-18 (3,849,034 parameters), together with representative literature methods (Table 2, Figure 6). Averaged over the four loads, FAL-CNN, SimpleCNN-65k, SimpleCNN-9k, and ResNet1D-18 attain 99.78%, 99.89%, 99.71%, and 99.83%, respectively. We report this candidly: on the saturated, abundant data CWRU benchmark, every compact model exceeds 99.7%, so full-data accuracy alone cannot distinguish architectures—and the parameter-matched plain CNN in fact scores 0.11 points higher than FAL-CNN. The justification for the frequency-aware design instead emerges under distribution shift: trained on one load and evaluated on unseen loads (Section 4.8), FAL-CNN retains (88.42 ± 2.66)% mean off-diagonal accuracy vs. (75.15 ± 2.35)% for SimpleCNN-65k (+13.27 points) and (73.73 ± 3.23)% for SimpleCNN-9k (+14.69 points), while matching a 59× larger ResNet-18 ((87.96 ± 2.78)%) at 5.6× fewer MACs. Frequency-masking and attribution analyses (Section 4.5) further show that the multi-scale filters respond specifically to the fault frequency bands. Under moderate training class imbalance, FAL-CNN maintains a macro-F1 above 99.2% (Section 4.7), and on the Paderborn real damage benchmark, it attains the best mean accuracy among the compared pipelines on unseen bearings (Section 4.9). Literature values (WDCNN 99.50%, TICNN 99.30%, 1D-ViT 99.90%) are single-run results under heterogeneous protocols and are provided for background only.

### 4.5. Ablation Study

**Figure 7:**
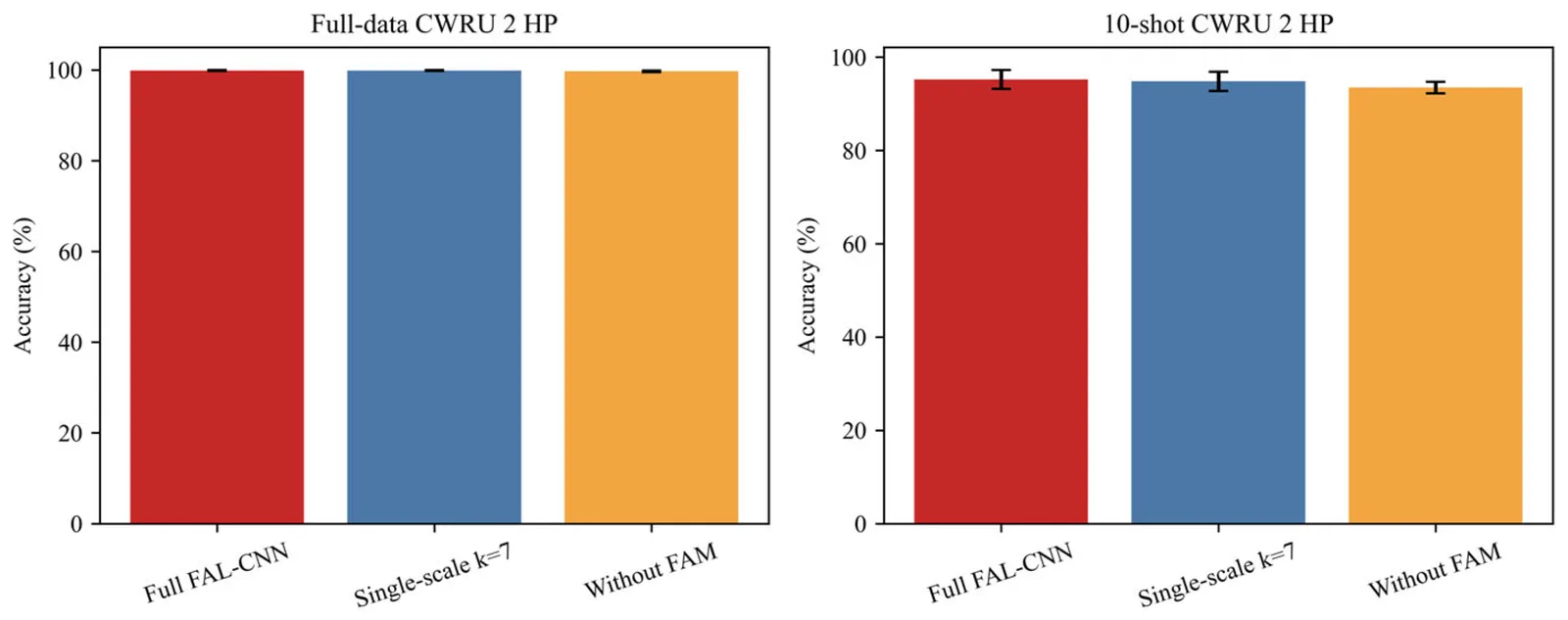
Component ablation on the CWRU 2 HP load (five seeds, mean ± SD). Full-data accuracy is saturated across variants. Under the 10-shot protocol, the full model shows a positive but statistically non-significant mean advantage over the single-scale and FAM-free variants.

To quantify the contribution of each component, we evaluate four variants on the 2 HP load (Table 3, Figure 7). All retrained variants use the leakage-controlled protocol with five random seeds, reported as mean ± standard deviation; the architecture-only variants C and D are retained from the original single-run protocol and are labeled legacy, since their parameter and MAC counts are protocol-independent while their accuracy numbers are not.

A (Full): The complete FAL-CNN, (99.88 ± 0.13)%.
B (w/o Frequency-Aware): The FAM is replaced by a single 1 × 1 convolution; accuracy is (99.70 ± 0.15)%, a 0.18-point drop, and a single-scale k = 7 variant of the FAM ties the full model at (99.88 ± 0.13)%—under the abundant data regime of CWRU, the multi-scale filter does not further lift full-data accuracy.
C (w/o Depthwise-Separable, legacy single-run): Standard convolutions replace the separable blocks; accuracy is 100.00%, but parameters jump to 146,082 (2.24×) and computation to 10.12 M MACs (20.24 M FLOPs; approximately 2.6× the full model), confirming the role of separable convolutions in controlling model size.
D (Time-Domain Input, legacy single-run): The raw 1024-sample segment is used instead of the 128-dim spectrum; accuracy stays at 99.72%, but computation rises to 30.91 M MACs (61.82 M FLOPs; approximately 8.0× the full model), confirming the efficiency gain of the spectral front-end.

The ablation yields two complementary findings. On the efficiency axis, the contributions are unambiguous and protocol-independent: the spectral front-end and the separable backbone reduce MACs by approximately 8.0× and 2.6×, respectively, at no accuracy penalty. On the accuracy axis, however, the full-data results of the retrained variants saturate within a 0.2-point band (99.70–99.88%), and even with the 10-shot protocol, the removal of the FAM costs only 1.73 points ((95.18 ± 2.05)% vs. (93.45 ± 1.25)%), with a paired 95% confidence interval of [−1.59, 5.05] that crosses zero. We therefore no longer claim a statistically separable per-module accuracy contribution. Instead, the masking and attribution analyses below (Table 4) provide direct evidence that the multi-scale filters, and not the backbone, carry the frequency-specific behavior, and Section 4.8 shows where this behavior translates into a large practical gain under operating-load shift.

**Figure 8:**
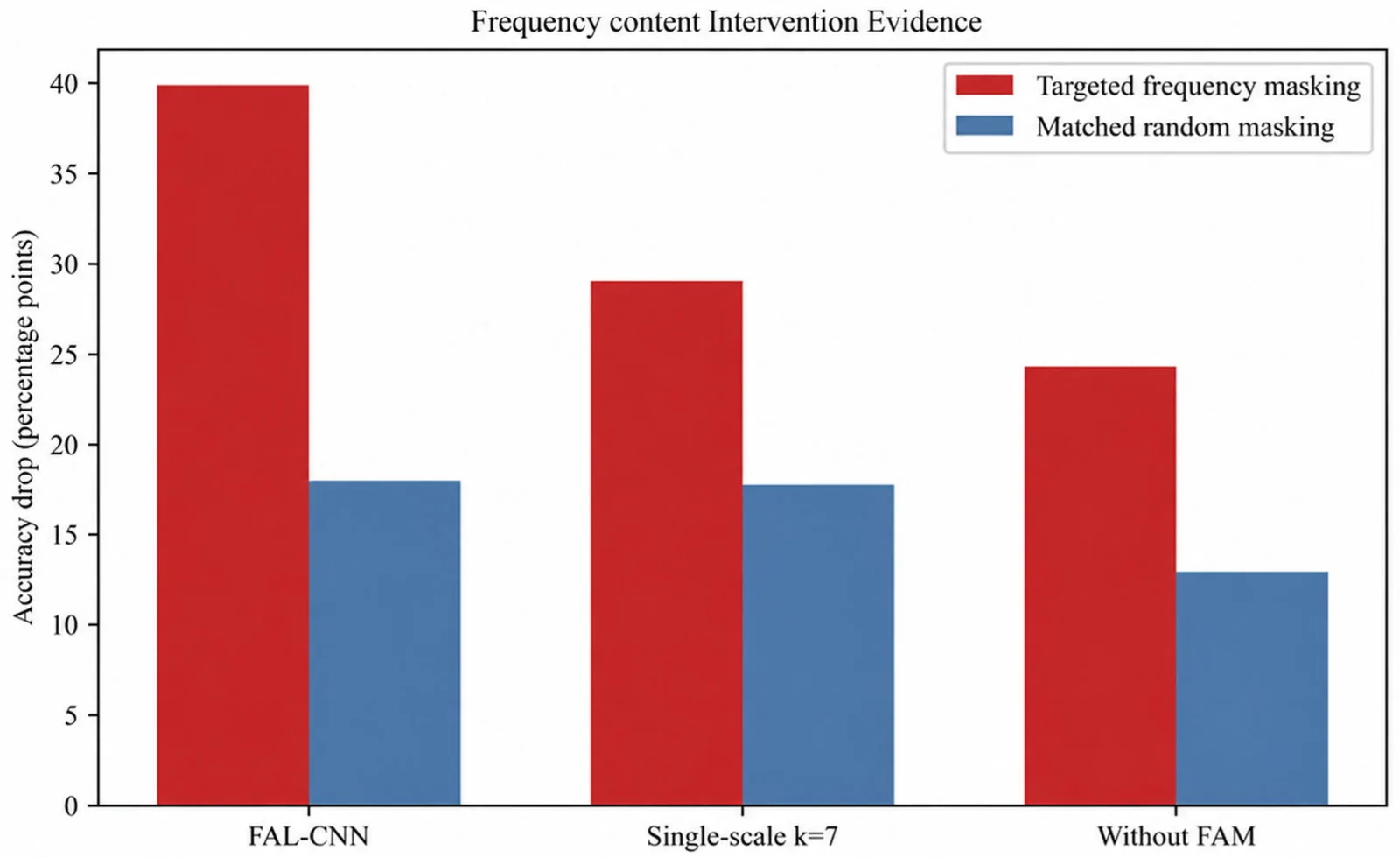
Frequency content intervention evidence on the CWRU 2 HP load (five seeds). Masking training-derived discriminative frequency bins causes a larger accuracy reduction than matched random masking. For FAL-CNN, the respective drops are 39.88 ± 7.58 and 17.97 ± 1.55 percentage points.

The saturated full-data regime cannot reveal how the frequency-aware module actually uses spectral information. We therefore probe the trained models directly with two complementary frequency-domain analyses on the 2 HP load, each repeated with five seeds (Table 4, Figure 8): (i) targeted masking—we zero the spectral bins covering the CWRU characteristic fault frequency band and measure the accuracy drop against a control that zeroes random bins of equal width; and (ii) attribution—we measure the Integrated Gradients (IG) attribution mass concentrated in the fault-band bins against the concentration over random bins.

The masking analysis provides direct evidence of frequency-specific dependence. For FAL-CNN, masking the fault frequency band drops accuracy by 39.88 ± 7.58 percentage points, whereas masking random bins of equal width drops it by only 17.97 ± 1.55 percentage points; the 21.91 ± 7.71 point difference is nearly twice that of the structural controls—the single-scale k = 7 variant (11.29 ± 8.02) and the FAM-free variant (11.39 ± 4.89). In other words, a substantial part of FAL-CNN’s decision behavior is specifically attributable to the fault frequency content rather than to generic spectral energy. The attribution analysis is consistent: IG attribution mass concentrates on the fault-band bins at 16.80% vs. 9.40% over random bins, an excess of +7.40 ± 0.51 points, roughly 1.4× the excess of the single-scale (+5.32 ± 0.87) and FAM-free (+5.28 ± 0.56) controls. Together, these results show that the multi-scale FAM learns responses that are both fault-frequency-specific and stronger than what a single-scale or FAM-free architecture provides, even though this difference does not translate into full-data accuracy on the saturated benchmark.

A separate single-run kernel-configuration scan under the legacy protocol (seed = 42) previously found all tested kernel sets within 99.72–100.00% on the full dataset; we retain those numbers only as legacy secondary evidence and no longer use them to argue for the (3, 7, 15) set. The repeated masking and attribution analyses above, together with the cross-load results in Section 4.8, constitute the primary evidence for the frequency-aware design.

### 4.6. Complexity and Edge Deployment

We export the trained 2 HP model to the Open Neural Network Exchange (ONNX) format via batch-normalization folding and a dynamic batch axis, producing a 288.8 KB file that stores all 65,122 weights in single-precision floating point (FP32) together with the computation graph. No post-training quantization, pruning, or weight sharing is applied, so this is the genuine FP32 footprint: the 65,122 FP32 weights alone occupy approximately 260 KB, and the remaining ≈ 29 KB accommodates the ONNX graph definition, operator metadata, and constant folding, comfortably within the 1 MB edge-storage budget. On the development CPU (ONNX Runtime 1.19.2, one intra-op and one inter-op thread, 100 warm-up plus 1000 timed iterations), single-sample inference takes a median of 0.17 ms (95th percentile (P95) 0.29 ms), and the complete preprocessing-plus-inference pipeline—z-score normalization (0.033 ms), real fast Fourier transform (RFFT) with log compression (0.015 ms), spectral resampling 513→128 (0.005 ms), min–max scaling and reshaping (0.012 ms), and ONNX inference (0.170 ms)—takes a median of 0.27 ms (P95 0.47 ms) end-to-end (Table 5). The maximum absolute difference between the PyTorch 2.2.2 and ONNX Runtime 1.19.2 outputs on the real test set is below 1 × 10^−5^, with identical predicted labels, confirming numerical fidelity across frameworks. These timings deliberately exclude data acquisition, disk I/O, window accumulation, communication, and OS scheduling, which are platform- and installation-specific. To relate the latency to the sensing cadence, note that at 12 kHz a 1024-sample window spans 85.3 ms, and the stride 256 schedule produces one window every 21.3 ms, so the 0.27 ms end-to-end cost consumes about 1.3% of the inter-window budget; batch inference sustains 3400–4700 samples/s. On the measured development CPU, this profile leaves substantial compute time headroom relative to the 21.3 ms inter-window interval. Whether equivalent real-time performance and sufficient headroom are achieved on an industrial gateway or microcontroller must be established by direct on-device measurements [16,18]. The ONNX graph can in principle be converted or compiled for backends such as TensorRT, OpenVINO, or TFLite Micro. Any latency, memory, or quantization benefit on those backends must be measured separately and is not claimed here.

We scope the deployment claims precisely. The measurements above were obtained on our development CPU and are reported as a reproducible baseline together with the released benchmark script bench_onnx_edge.py, so they can be re-run on any target device. We do not present projected latencies for specific edge boards: representative on-device measurements on physical edge boards were not available in our evaluation environment, and we consider such on-board profiling—together with the ongoing field deployment at the Taizhou rail transit maintenance site—a required step before production use. The deployment argument instead rests on three hardware-independent facts: the model’s arithmetic cost (3.88 M MACs; 7.76 M FLOPs), its memory footprint (288.8 KB FP32), and its measured sub-millisecond preprocessing-plus-inference latency on the development CPU. Operationally, we adopt a center–edge maintenance architecture: models are trained and updated on a central server or maintenance workstation, where class weighting, resampling, and other training-time remedies are applied, and only the fixed inference graph is deployed to the edge node, which performs preprocessing and diagnosis without any training computation. This division keeps the edge node’s compute and energy budget dominated by sensing and inference and allows model updates to be validated centrally before rollout.

### 4.7. Evaluation Under Limited Labels and Class Imbalance

**Figure 9:**
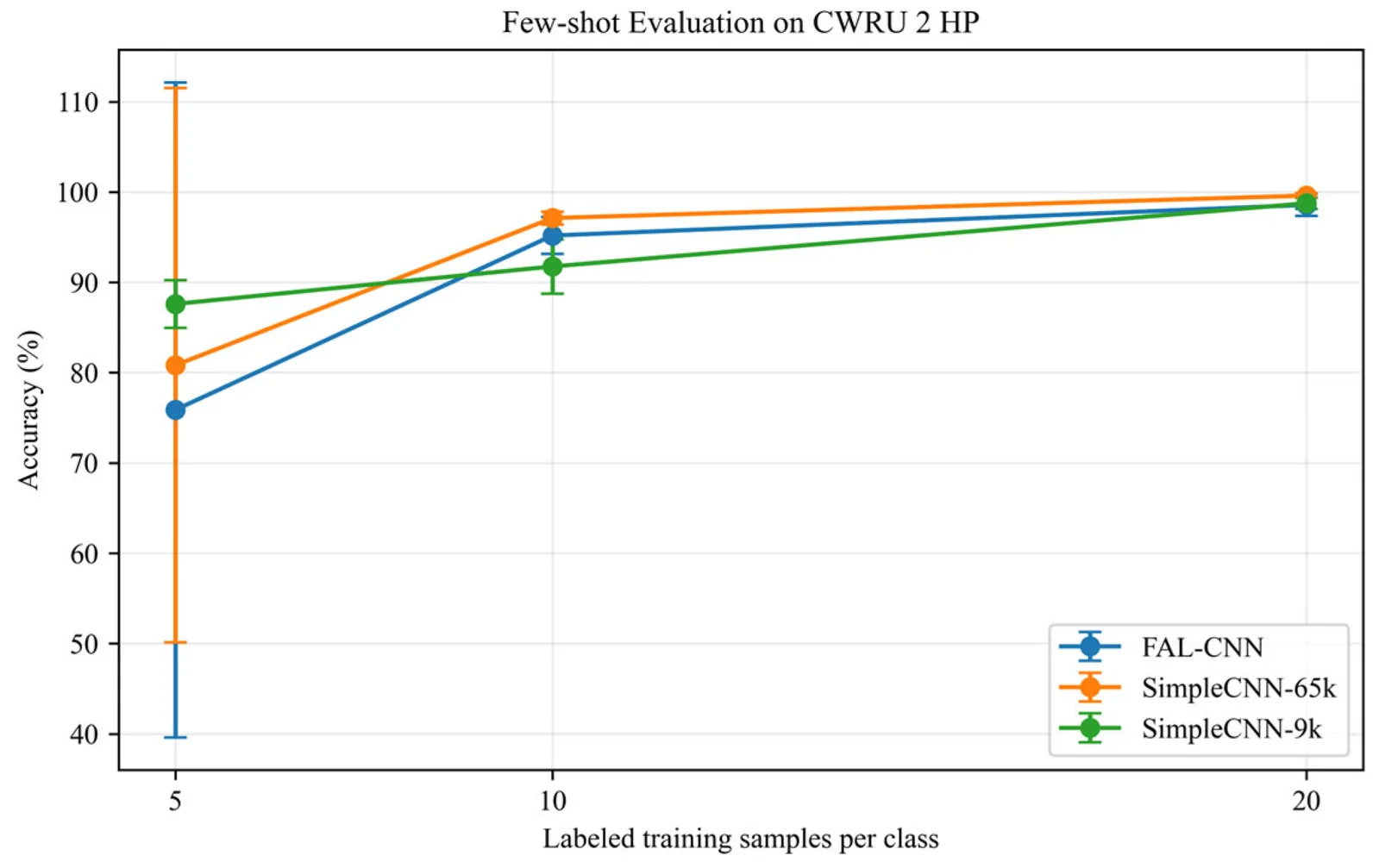
Few-shot evaluation on the CWRU 2 HP load with 5, 10, and 20 labeled samples per class (five seeds, mean ± SD). Under the 5-shot protocol, FAL-CNN and SimpleCNN-65k show large seed variation, whereas SimpleCNN-9k is comparatively stable; by 20-shot, all three models exceed 98.5% accuracy.

Edge scenarios often suffer from scarce labeled data because faults are inherently rare and labeling requires inspection records or expert confirmation. We retrain FAL-CNN, SimpleCNN-65k, and SimpleCNN-9k on the 2 HP load with k ∈ {5, 10, 20} labeled samples per class, five seeds each, keeping the failed runs in the averages (Figure 9; few-shot rows of Table 5). The results are sobering, and we report them without selection: under the 5-shot protocol, FAL-CNN reaches only (75.88 ± 36.26)%,, SimpleCNN-65k (80.82 ± 30.70)%, and SimpleCNN-9k (87.61 ± 2.63)%—the two larger models are destabilized by failed runs (SD ≈ 31–36 points), the smallest model is comparatively stable, but the inconsistent ordering does not support a general claim of reliable 5-shot deployment. Under the 10-shot protocol, the ordering partially reverses (FAL-CNN (95.18 ± 2.05)%, SimpleCNN-65k (97.12 ± 0.71)%, SimpleCNN-9k 91.76% ± 3.00), and under the 20-shot protocol, all three models exceed 98.5% ((98.58 ± 1.22)%, (99.61 ± 0.28)%, (98.76 ± 0.64)%). We therefore retract the earlier claim of a stable few-shot advantage for the frequency-aware design: under the leakage-controlled, multi-seed protocol, the few-shot differences between the three architectures are small, seed-dependent, and inconsistent across k. The practical implications are instead that (i) roughly ten labels per class already suffice for any of these compact spectral-input models to exceed 95% on this benchmark, substantially lowering the cold-start cost, and (ii) architecture selection under label scarcity cannot be justified by few-shot accuracy alone; the reproducible architectural benefit of the frequency-aware design lies in cross-load transfer (Section 4.8) and frequency-specific behavior (Section 4.5), not in few-shot learning.

A more realistic data-scarcity pattern in predictive maintenance is class imbalance: normal vibration accumulates continuously, whereas fault labels are scarce and expensive to confirm. We therefore construct imbalanced training sets by retaining all 319 normal windows per class and subsampling each of the nine fault classes to 64 or 32 windows, corresponding to normal-to-fault ratios of approximately 5:1 and 10:1 per fault class; the validation and test sets remain balanced and unchanged, so macro-averaged metrics directly measure the retention of fault-class recognition. All methods use five seeds and identical splits. The compared approaches are the three CNNs with standard cross-entropy (CE) and with class-weighted CE, classical baselines on the same spectral features (decision tree, random forest, SVM), and SMOTE-Tomek resampling combined with SVM. Resampling and loss weighting are applied only to the training set and only at the central training stage—they change nothing in the edge inference graph.

The results (Table 6) show that the spectral representation is robust to moderate training imbalance: FAL-CNN retains a macro-F1 of (99.27 ± 0.80)% (CE) and (99.30 ± 0.33)% (weighted CE) at 10:1, and (99.76 ± 0.14)%/(99.88 ± 0.17)% at 5:1; SimpleCNN-65k behaves similarly (99.76–99.85 at both ratios), SimpleCNN-9k drops slightly (98.97–99.12 at 10:1), and the classical baselines remain strong (SVM 99.58, SMOTE-Tomek SVM 99.55, random forest 99.94, decision tree 95.44 at 10:1). Two honest observations follow. First, at these ratios with balanced evaluation, no architecture collapses, and the differences between methods are small—imbalance alone does not separate architectures. Second, class-weighted CE provides a small but consistent gain for FAL-CNN at zero inference cost, which fits the center-train/edge-infer workflow described in Section 4.6. These experiments scope, rather than oversell, the imbalance robustness of the proposed model; more severe imbalance, imbalance at evaluation time, and joint cross-dataset scarcity conditions remain future work.

Finally, to connect with unsupervised alternatives for the scarce-label regime, we evaluate a one-class anomaly-screening baseline: an isolation forest operating on the same 128-dimensional spectral features achieves (99.58 ± 0.17)% accuracy and (99.76 ± 0.09)% F1 for the binary healthy-vs.-fault task, with an ROC-AUC of 1.00. This confirms that the fixed spectral representation itself is highly separable and that isolation forests are a viable low-cost pre-screen; the multi-class CNN remains necessary when the fault location and severity must be identified for maintenance planning.

### 4.8. Robustness Under Realistic Industrial Degradations

The near-parity among the compact models on the clean, abundant data CWRU benchmark (Section 4.4) is a saturated regime artifact: a minimal convolutional stack already extracts the easily separable spectral cues. Deployment-relevant scenarios can include noisy sensors and machines operating at loads unseen during training. Section 4.7 has already shown that limited labels and moderate imbalance also fail to separate architectures. Here, we stress-test the models under the two remaining deployment-relevant perturbations—raw-signal noise and operating-load shift—and evaluate whether the frequency-aware design retains an advantage under these conditions.

**Figure 10:**
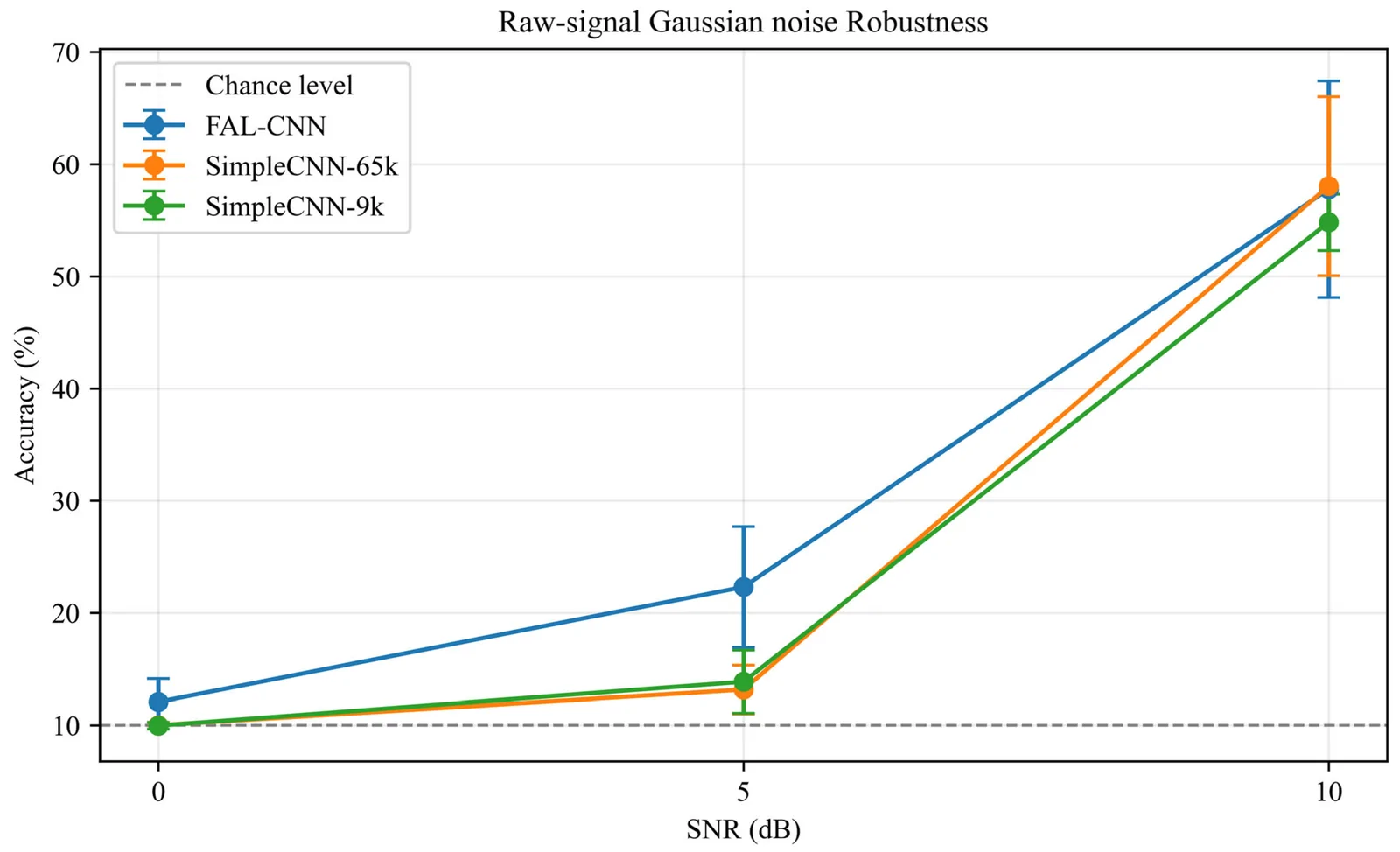
Robustness to additive white Gaussian noise injected into the unstandardized raw time-domain test windows before spectral preprocessing (CWRU 2 HP, five seeds, mean ± SD). FAL-CNN degrades less than both CNN controls at 5 dB, whereas all models approach chance-level performance at 0 dB.

Raw-signal broadband noise. We inject additive white Gaussian noise onto the raw, un-normalized 1024-sample vibration windows of the 2 HP test set—before z-score normalization, the FFT, and all subsequent spectral processing—so that the full front-end operates on genuinely corrupted signals. The same noise realizations are shared by all models at each signal-to-noise ratio (SNR), and all models are trained only on clean data (five seeds; Table 7, Figure 10). At 10 dB, the three CNNs are statistically indistinguishable (FAL-CNN (57.76 ± 9.64)%, SimpleCNN-65k (58.03 ± 7.98)%, SimpleCNN-9k (54.80 ± 2.53)%). At 5 dB, FAL-CNN retains (22.29 ± 5.38)% vs. (13.15 ± 2.16)% and (13.85 ± 2.80)% for the two plain CNNs—advantages of 9.14 and 8.44 points. At 0 dB all models collapse toward the 10% chance level (FAL-CNN (12.06 ± 2.08)%). We scope these results explicitly: this is a controlled stress test of sensitivity to Gaussian corruption, not a validated industrial-noise robustness claim; FAL-CNN degrades less than the two CNN controls at 5 dB, while no evaluated model remains usable at 0 dB, and colored noise, impulsive interference, and coupled vibration from adjacent machinery are not covered.

**Figure 11:**
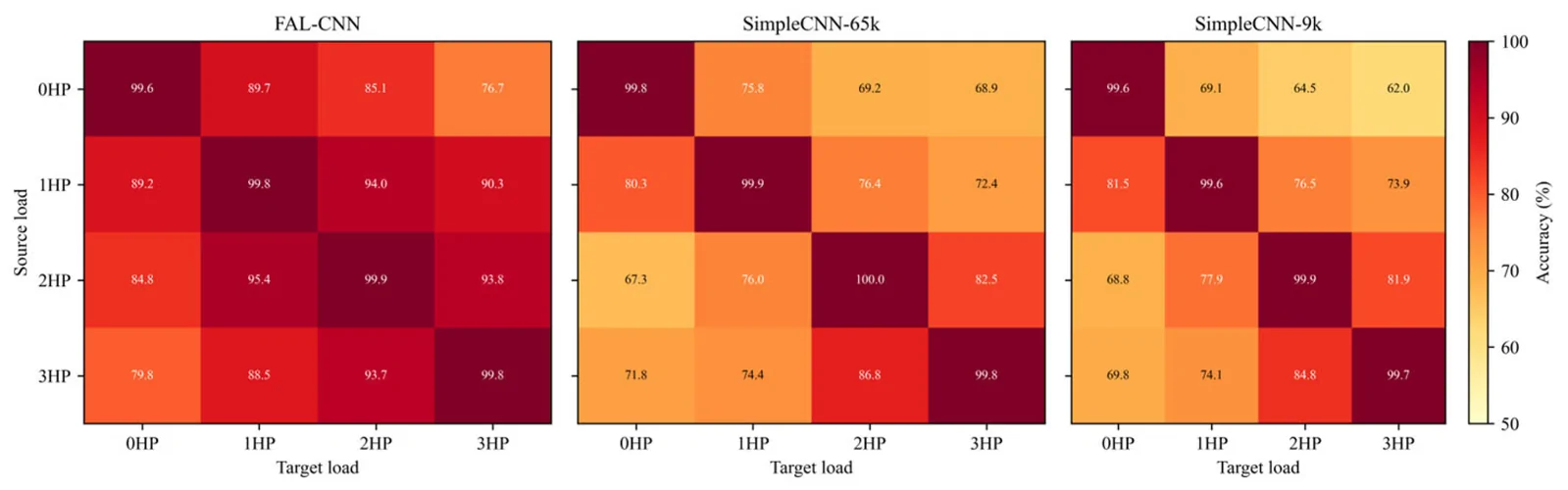
Source-only cross-load generalization across all CWRU source-target load combinations (five-seed means). Mean off-diagonal accuracy is 88.42 ± 2.66% for FAL-CNN, 75.15 ± 2.35% for SimpleCNN-65k, and 73.73 ± 3.23% for SimpleCNN-9k.

Cross-load domain shift. We perform the stricter and, as it turns out, decisive test: train on one load and evaluate on an unseen load, repeating all 4 × 4 source–target combinations with five seeds (Table 8, Figure 11). FAL-CNN retains a mean off-diagonal accuracy of (88.42 ± 2.66)%, while the parameter-matched SimpleCNN-65k drops to (75.15 ± 2.35)% and SimpleCNN-9k to (73.73 ± 3.23)%—gaps of 13.27 and 14.69 points. Two observations sharpen the attribution. First, capacity alone does not explain the gap: enlarging the plain CNN from 9k to 65k parameters buys only 1.42 points (73.73 → 75.15), whereas the frequency-aware design at the same 65k budget buys 13.27 points over its parameter-matched twin. Second, FAL-CNN statistically matches the 59× larger ResNet1D-18 ((87.96 ± 2.78)%) at 5.6× fewer MACs, and while only the spectral-feature SVM reaches a higher mean (89.61%), 1.19 percentage points above FAL-CNN, the decision tree collapses ((55.91 ± 0.91)%). Under the evaluated protocol, the fixed spectral front-end and multi-scale processing are associated with better source-only cross-load transfer than the two plain CNN controls; the comparison supports an architectural effect beyond parameter count, while it does not isolate a single component as the sole cause.

Taken together, these results reframe the cost–performance comparison. The 12.9× larger MAC budget of FAL-CNN relative to SimpleCNN-9k buys no measurable full-data accuracy—the parameter-matched SimpleCNN-65k in fact scores 0.11 points higher on the saturated benchmark—but under the evaluated load-shift and noise conditions it provides a 13.27-point reliability margin under load shift, a 9.14-point margin under 5 dB raw-signal noise, and measurably frequency-specific decision behavior (Section 4.5). The plain CNNs are computationally cheaper, but their accuracy is less stable under the evaluated load shift and 5 dB Gaussian noise settings. Conversely, the collapse of all models at 0 dB noise and the instability of FAL-CNN and SimpleCNN-65k under the 5-shot protocol, together with the inconsistent model ordering, bounds the claim: the advantage of the frequency-aware design is conditional on the input retaining usable signal content and on at least moderate label availability.

### 4.9. External Validation on Real Damage Data (Paderborn)

All evaluations so far use the CWRU benchmark, whose faults are artificially induced by electro-discharge machining. To test the model on real damage and on bearings never seen during training, we additionally evaluate on the Paderborn University bearing dataset under the N15_M07_F10 operating condition, which contains 15 bearings: five healthy, five with real inner-race damage, and five with real outer-race damage, where the damage resulted from accelerated lifetime testing rather than machining. This is a 3-class task on a different acquisition platform with a different damage formation mechanism; windows of 1024 samples with stride 1024 (no overlap) are extracted per bearing. We use a bearing-level nested protocol: an outer five-fold split over bearings guarantees that the three test bearings of each fold participate in neither training nor model selection; within each training set, an inner four-fold cross-validation selects the input representation (native full-band, legacy 12 kHz downsampled, or native envelope) and hyperparameters under an identical budget for every model; the final model of each fold is trained with three seeds and evaluated on the unseen bearings. We report the mean of the seed means per fold and then the mean ± standard deviation across the five folds, so the reported spread reflects bearing-to-bearing variation.

**Figure 12:**
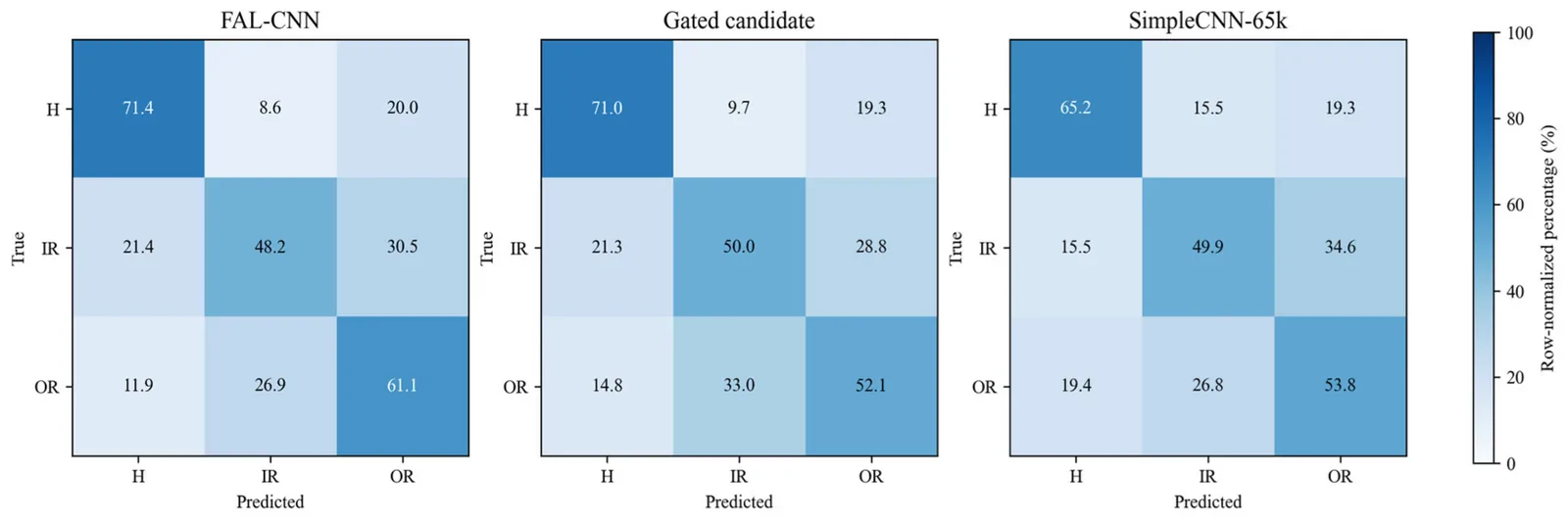
Row-normalized confusion matrices aggregated over the final Paderborn held-out-bearing outer-fold and seed predictions under the nested selection protocol. Results are shown for FAL-CNN, the validation-selected gated candidate, and SimpleCNN-65k; the matrices illustrate the remaining difficulty in distinguishing real inner-race and outer-race damage on unseen bearings.

The results (Table 9, Figure 12) provide complementary but bounded evidence. The original FAL-CNN attains the highest mean accuracy ((59.86 ± 18.86)%) and macro-F1 ((55.50 ± 19.63)%) among the three compared pipelines, ahead of the parameter-matched SimpleCNN-65k ((57.47 ± 24.86)% accuracy; (54.70 ± 24.43)% macro-F1) and a validation-only gated variant of FAL-CNN ((56.15 ± 22.84)%; macro-F1 52.44 ± 22.45). The paired accuracy difference of +2.40 points over SimpleCNN-65k has an approximate 95% confidence interval of [−7.62, 12.41], which crosses zero: the mean advantage is not statistically significant. The fold-level spread is large—FAL-CNN’s fold accuracies range from near the 33.3% three-class chance level (32.86%) to 84.19%, consistent with the strong bearing-to-bearing variation typical of real damage data.

We interpret these results deliberately conservatively. The Paderborn experiment shows that, under an identical selection budget and a strict unseen bearing protocol, the proposed pipeline remains competitive on real damage data from a different platform, extending the evidence base beyond CWRU. It does not establish a statistically significant advantage, nor cross-condition generalization on this dataset (the operating condition is fixed), nor field readiness. Together with the controlled CWRU cross-load results (Section 4.8), this external validation broadens the evidence beyond artificially damaged bearings while showing that unseen bearing generalization remains unresolved.

### 4.10. Discussion and Summary of Findings

Taken together, the experiments support five conclusions. First, the fixed spectral front-end is the single most important efficiency lever: the legacy time-domain variant reaches comparable accuracy only with an approximately 8.0× larger MAC budget, and the complete FAL-CNN pipeline degrades less than the two plain CNN controls at 5 dB in Section 4.8. Second, the frequency-aware module is not an accuracy lever on the saturated CWRU benchmark—removing it costs only 0.18 points at full data and a non-significant 1.73 points under the 10-shot protocol—but it is the transfer and frequency-specificity lever: +13.27 points of mean cross-load accuracy over a parameter-matched plain CNN, and a targeted-masking sensitivity gap (21.91 points) roughly twice that of structural controls. Third, depthwise-separable convolution is the parameter lever: replacing it (legacy variant C) more than doubles parameters and MACs at negligible accuracy gain. Fourth, the exported model is compact (288.8 KB), and the development-computer measurements are 0.17 ms for ONNX inference and 0.27 ms for preprocessing plus inference; these values establish implementation feasibility on the measured CPU but are not physical edge-device results. Fifth, external validation on real damage data with unseen bearings (Paderborn) yields the best mean among the compared pipelines ((59.86 ± 18.86)%) but with a confidence interval that crosses zero, bounding the claim to competitiveness rather than superiority. The value proposition of this paper is therefore reliability at matched cost under distribution shift, not saturated benchmark accuracy.

The limitations revealed by the revised evaluation are equally clear. Under the 5-shot protocol, FAL-CNN and SimpleCNN-65k are unstable (seed standard deviations of approximately 31–36 points), whereas SimpleCNN-9k is comparatively stable but does not establish a consistent architectural ordering; consequently, the evaluated models support more reliable conclusions from approximately ten labels per class onward. Raw-signal noise below 5 dB collapses all models toward chance, and colored or impulsive industrial interference is untested. Cross-load transfer, while large (+13.27 points), is still evaluated within one benchmark family, and the Paderborn unseen bearing results show high bearing-to-bearing variance (SD ≈ 19–25 points) with no statistically significant advantage. The per-load training protocol means a new operating load requires retraining, and the deployment measurements come from a development CPU rather than representative edge boards; on-device profiling and the ongoing Taizhou rail transit field deployment are required before production claims. These limitations define the honest deployment envelope of the method.

To facilitate adoption and fair comparison, we will release the source code, the trained weights, the exported ONNX model, the benchmark script, and the exact preprocessing, splitting, and training configurations used in this study. All CWRU results use five fixed seeds (0, 1, 2, 3, 42) under a leakage-controlled time-blocked protocol shared by every compared model; the Paderborn study uses a bearing-level nested protocol with three seeds. Legacy single-run numbers are explicitly labeled wherever they are retained. From an engineering standpoint, the method imposes no exotic dependencies: the spectral front-end is a handful of linear and element-wise operations, the backbone uses standard convolution primitives that are widely supported by major inference engines, and the exported model runs on ONNX Runtime without a vendor-specific toolchain. This facilitates integration into existing condition monitoring software while leaving deployment on resource-constrained target hardware for direct validation.

## 5. Conclusions

This paper presented FAL-CNN, a frequency-aware lightweight convolutional network for edge-enabled intelligent bearing fault diagnosis and health management. By co-designing a fixed 128-dimensional spectral front-end and a depthwise-separable backbone, the model achieves a 99.78% mean accuracy across four CWRU operating loads under a leakage-controlled, five-seed protocol, while requiring only 65,122 parameters, 3.88 M MACs (7.76 M FLOPs), 0.17 ms single-thread CPU inference, and a 288.8 KB ONNX footprint. Because full-data accuracy saturates for all compact models, the revised evaluation deliberately locates the model’s value where architecture matters: a +13.27-point cross-load margin over a parameter-matched plain CNN, frequency-masking and attribution evidence that the multi-scale filters target fault frequency bands, a macro-F1 above 99.2% under 10:1 training imbalance, and the best mean accuracy ((59.86 ± 18.86)%) on the Paderborn real damage benchmark with strict unseen bearing validation—alongside reported limits under the 5-shot protocol, 0 dB noise, and unseen bearing variance. The latency measurements were obtained only on the development CPU and do not constitute validation on a physical edge device. Future work will (i) validate on additional machines, cross-dataset transfers, and varying-speed conditions via order tracking; (ii) deploy and profile the model on physical microcontrollers and edge boards within the ongoing Taizhou field program; (iii) investigate noise-adaptive or learnable front-ends for severe-SNR conditions; and (iv) extend the health management workflow beyond classification toward degradation assessment and remaining-useful-life estimation.

## Figures and Tables

**Table 1:** Case Western Reserve University (CWRU) 12 kHz Drive End bearing dataset composition under the leakage-controlled protocol (identical for all four loads). Each recording is split into continuous-record time blocks before window extraction (L = 1024, stride 256) with a 1024-sample isolation gap between blocks; every class contributes 319 training, 66 validation, and 66 test windows per load, and the same partitioning is used by every compared model.

Class	Fault Type	Train	Val	Test
Normal	Normal	319	66	66
IR007	Inner race 7 mil	319	66	66
IR014	Inner race 14 mil	319	66	66
IR021	Inner race 21 mil	319	66	66
B007	Ball 7 mil	319	66	66
B014	Ball 14 mil	319	66	66
B021	Ball 21 mil	319	66	66
OR007	Outer race 7 mil	319	66	66
OR014	Outer race 14 mil	319	66	66
OR021	Outer race 21 mil	319	66	66

**Table 2:** Controlled comparison on CWRU (12k Drive End, four loads, five seeds, identical time-blocked splits). Accuracy is the four-load mean of the five-seed means. Literature accuracies are single-run best results under heterogeneous protocols, listed for background only and not directly comparable. “N/R” denotes not reported.

Method	Accuracy (%)	Params	MACs	Source
FAL-CNN (Ours)	99.78	65,122	3.88 M	Self-run
SimpleCNN-65k	99.89	65,090	2.30 M	Self-run
SimpleCNN-9k	99.71	8714	0.30 M	Self-run
ResNet1D-18	99.83	3,849,034	21.79 M	Self-run
WDCNN	99.50	N/R	N/R	Literature
TICNN	99.30	N/R	N/R	Literature
1D-ViT	99.90	4,130,000	N/R	Literature

Note: the literature accuracies (WDCNN [5], TICNN [7], 1D-ViT [9]) are the original authors’ best reported single-run results, obtained under different data splits, preprocessing, and protocols; they are listed for context and are not directly comparable with the five-seed controlled means reported here.

**Table 3:** Ablation study on the 2 HP load. Retrained variants (A, B) use the leakage-controlled protocol with five seeds (mean ± standard deviation (SD)); variants C and D are legacy single-run results (seed = 42) whose parameter and MAC counts are protocol-independent. Full-data accuracies saturate within a 0.2-point band, and the 10-shot frequency-aware module (FAM) gap of 1.73 points has a 95% confidence interval (CI) of [−1.59, 5.05] (not significant); the efficiency contributions are unambiguous.

Variant	Full-Data Acc. (%)	10-Shot Acc. (%)	Params	MACs	Protocol
A_Full	99.88 ± 0.13	95.18 ± 2.05	65,122	3.88 M	5 seeds
B_wo_FAM	99.70 ± 0.15	93.45 ± 1.25	59,978	3.22 M	5 seeds
C_wo_DepthwiseSep	100.00	—	146,082	10.12 M	Legacy single-run
D_TimeDomain	99.72	—	65,122	30.91 M	Legacy single-run

**Table 4:** Frequency-domain masking and attribution evidence for the frequency-aware module (2 HP load, five seeds, mean ± SD). “Targeted-mask drop” is the accuracy drop when the fault frequency band is zeroed; “random-mask drop” uses random bins of equal width; their difference isolates frequency-specific dependence. “IG excess” is the excess of integrated gradients attribution concentration on fault-band bins over random bins (percentage points).

Variant	Targeted-Mask Drop (%)	Random-Mask Drop (%)	Difference (pp)	IG Excess (pp)
FAL-CNN (multi-scale, proposed)	39.88 ± 7.58	17.97 ± 1.55	21.91 ± 7.71	+7.40 ± 0.51
Single-scale k = 7	29.03 ± 13.21	17.74 ± 6.81	11.29 ± 8.02	+5.32 ± 0.87
w/o FAM	24.30 ± 4.58	12.92 ± 6.58	11.39 ± 4.89	+5.28 ± 0.56

**Table 5:** Complexity and edge-oriented implementation profile of FAL-CNN. Latency figures are medians (P95 in parentheses) on the development CPU with ONNX Runtime 1.19.2 (single intra-op thread; 100 warm-up + 1000 timed runs); the end-to-end figure includes the full spectral front-end and excludes acquisition, disk, communication, and scheduling. Few-shot accuracies are five-seed means on the 2 HP load.

Metric	Value
Parameters	65,122
MACs/FLOPs	3,878,528/7,757,056
ONNX inference latency (single sample)	0.17 ms (P95 0.29 ms)
End-to-end pipeline latency (incl. front-end)	0.27 ms (P95 0.47 ms)
Model file size (.onnx)	288.8 KB
Batch throughput (ONNX Runtime)	3400–4700 samples/s
5-shot accuracy (2 HP)	(75.88 ± 36.26)%
10-shot accuracy (2 HP)	(95.18 ± 2.05)%
20-shot accuracy (2 HP)	(98.58 ± 1.22)%

**Table 6:** Performance under training class imbalance (macro-F1 (%), five seeds, mean ± standard deviation (SD); balanced validation and test sets). Ratios are normal-to-fault per fault class; all methods share identical splits and spectral inputs. Synthetic Minority Oversampling Technique with Tomek links (SMOTE-Tomek) resampling and loss weighting are training-time-only operations and do not alter the edge inference graph.

Method	5:1	10:1
FAL-CNN (CE)	99.76 ± 0.14	99.27 ± 0.80
FAL-CNN (weighted CE)	99.88 ± 0.17	99.30 ± 0.33
SimpleCNN-65k (CE)	99.76 ± 0.14	99.76 ± 0.17
SimpleCNN-65k (weighted CE)	99.88 ± 0.20	99.85 ± 0.11
SimpleCNN-9k (CE)	99.73 ± 0.29	98.97 ± 0.20
SimpleCNN-9k (weighted CE)	99.76 ± 0.14	99.12 ± 0.17
SVM (spectral features)	99.70 ± 0.15	99.58 ± 0.17
SMOTE-Tomek + SVM	99.70 ± 0.21	99.55 ± 0.19
Random forest	100.00 ± 0.00	99.94 ± 0.14
Decision tree	97.04 ± 0.86	95.44 ± 1.23

**Table 7:** Robustness against broadband Gaussian noise injected on the raw vibration signal before the spectral front-end (2 HP test set, five seeds, mean ± SD). All models are trained on clean data only and share identical noise realizations at each SNR. The clean row also reports the five-seed mean ± SD under the same 2 HP split. At 5 dB, FAL-CNN retains a 9.14/8.44-point margin over SimpleCNN-65k/9k; at 0 dB, all models collapse toward the chance level.

Condition	FAL-CNN (%)	SimpleCNN-65k (%)	SimpleCNN-9k (%)
Clean (full data, 2 HP)	99.88 ± 0.13	99.97 ± 0.07	99.91 ± 0.14
Raw-signal noise 10 dB	57.76 ± 9.64	58.03 ± 7.98	54.80 ± 2.53
Raw-signal noise 5 dB	22.29 ± 5.38	13.15 ± 2.16	13.85 ± 2.80
Raw-signal noise 0 dB	12.06 ± 2.08	10.00 ± 0.04	9.96 ± 0.30

**Table 8:** Cross-load generalization (accuracy (%), five-seed means). Each three-row block uses the indicated source load for training and reports FAL-CNN, SimpleCNN-65k, and SimpleCNN-9k separately across the four target loads. Mean off-diagonal accuracy is (88.42 ± 2.66)% for FAL-CNN, (75.15 ± 2.35)% for SimpleCNN-65k, and (73.73 ± 3.23)% for SimpleCNN-9k. The respective gaps of 13.27 and 14.69 percentage points support an architectural effect beyond parameter count under the evaluated protocol, without isolating a single component as the sole cause.

Trained Load	Model	Test: 0 HP	Test: 1 HP	Test: 2 HP	Test: 3 HP
0 HP	FAL-CNN	99.64	89.73	85.06	76.70
	CNN-65k	99.85	75.85	69.15	68.91
	CNN-9k	99.61	69.12	64.52	62.00
1 HP	FAL-CNN	89.21	99.76	93.97	90.33
	CNN-65k	80.30	99.91	76.42	72.39
	CNN-9k	81.52	99.58	76.45	73.91
2 HP	FAL-CNN	84.85	95.36	99.88	93.85
	CNN-65k	67.33	75.97	99.97	82.55
	CNN-9k	68.76	77.91	99.91	81.88
3 HP	FAL-CNN	79.82	88.45	93.67	99.82
	CNN-65k	71.82	74.39	86.76	99.82
	CNN-9k	69.79	74.12	84.82	99.73

**Table 9:** External validation on the Paderborn real damage dataset (N15_M07_F10, 3-class, bearing-level five-fold nested cross-validation, three seeds per fold, input representation selected by inner cross-validation under an identical budget). Accuracy and macro-F1 are means across outer folds ± SD across folds. Parameter counts are for the three-class implementations and are therefore lower than the corresponding ten-class CWRU counts. The gated candidate is selected using validation data only, without access to target-test labels. The approximate paired confidence interval for FAL-CNN vs. SimpleCNN-65k is reported in the accompanying text.

Model	Params	Accuracy (%)	Macro-F1 (%)
FAL-CNN (original)	64,667	59.86 ± 18.86	55.50 ± 19.63
FAL-CNN (gated candidate)	65,007	56.15 ± 22.84	52.44 ± 22.45
SimpleCNN-65k	63,795	57.47 ± 24.86	54.70 ± 24.43

## Data Availability

Publicly available data were analyzed in this study. The CWRU Bearing Data Center dataset is available at https://engineering.case.edu/bearingdatacenter (accessed on 13 September 2026). No new primary dataset was created in this study. The source code, trained model weights, exported ONNX model, and intermediate processed data are not publicly available.
